# The Natural Polypeptides as Significant Elastase Inhibitors

**DOI:** 10.3389/fphar.2020.00688

**Published:** 2020-06-05

**Authors:** Shabir Ahmad, Muhammad Saleem, Naheed Riaz, Yong Sup Lee, Reem Diri, Ahmad Noor, Diena Almasri, Alaa Bagalagel, Mahmoud Fahmi Elsebai

**Affiliations:** ^1^Department of Chemistry, Baghdad-ul-Jadeed Campus, The Islamia University of Bahawalpur, Bahawalpur, Pakistan; ^2^Department of Chemistry, Post-Graduate College, Bahawalpur, Pakistan; ^3^Department of Life and Nanopharmaceutical Sciences & Medicinal Chemistry Laboratory, Department of Pharmacy, College of Pharmacy, Kyung Hee University, Seoul, South Korea; ^4^Department of Pharmacy Practice, Faculty of Pharmacy, King Abdulaziz University, Jeddah, Saudi Arabia; ^5^Department of Natural Products and Alternative Medicine, Faculty of Pharmacy, University of Tabuk, Tabuk, Saudi Arabia; ^6^Department of Pharmacognosy, Faculty of Pharmacy, Mansoura University, Mansoura, Egypt

**Keywords:** elastases, natural products, polypeptides, anti-inflammatory, marine natural products

## Abstract

Human neutrophil elastase (HNE) is a major cause of the destruction of tissues in cases of several different chronic andinflammatory diseases. Overexpression of the elastase enzyme plays a significant role in the pathogenesis of various diseases including chronic obstructive pulmonary disease (COPD), acute respiratory distress syndrome, rheumatoid arthritis, the rare disease cyclic hematopoiesis (or cyclic neutropenia), infections, sepsis, cystic fibrosis, myocardial ischemia/reperfusion injury and asthma, inflammation, and atherosclerosis. Human neutrophil elastase is secreted by human neutrophils due to different stimuli. Medicine-based inhibition of the over-activation of neutrophils or production and activity of elastase have been suggested to mend inflammatory diseases. Although the development of new elastase inhibitors is an essential strategy for treating the different inflammatory diseases, it has been a challenge to specifically target the activity of elastase because of its overlapping functions with those of other serine proteases. This review article highlights the reported natural polypeptides as potential inhibitors of elastase enzyme. The mechanism of action, structural features, and activity of the polypeptides have also been correlated wherever they were available.

## Introduction

The regulation of immune response and controlling inflammation are the customary roles of human neutrophil cells. Along with other serine proteases, human neutrophils secrete HNE, which is a 29 kDa serine proteases of the chymotrypsin family. HNE contains a charge relay system consisting of the catalytic triad of aspartate, serine, and histidine residues that are dispersed throughout the polypeptide. Neutrophil elastase is closely related to other cytotoxic immune serine proteases, such as cathepsin G, proteinase 3, and the granzymes (Thomas et al., 2014; Hilhorst et al., 2015). It has a broad substrate specificity. For example, the intracellular HNE breaks down pathogenic proteins, whereas the extracellular HNE helps in the migration of neutrophils to the inflammation sites through the degradation of the extracellular matrix proteins. HNE has the ability to degrade the body's own cellular matrix as well as proteins that are foreign to the body. HNE also conducts proteolysis and plays a significant role in several biological processes. Despite the positive attributes, overproduction and uncontrolled functioning of elastase may produce devastating effects and cause serious damage to the host (Crocetti et al., 2019).

Under normal conditions, the activity of HNE is controlled by endogenous inhibitors including secretory leukocyte proteinase inhibitor, α1-antitrypsin (α1-AT), α2- macroglobulin, and elafin, but excessive and uncontrolled activity of HNE can cause serious damage to the tissues resulting in COPD, cyclic hematopoiesis, pulmonary emphysema (the loss of lungs elasticity), rheumatoid arthritis, pancreatitis, cystic fibrosis, psoriasis and bullous dermatoses, asthma, and systemic inflammatory response syndrome (Horwitz et al., 1999; Elsebai et al., 2012; Crocetti et al., 2019; Thulborn et al., 2019). HNE also localizes to Neutrophil extracellular traps (NETs) through its high DNA affinity, an unusual property for serine proteases. Studies have established that NETs are associated with increased lung injury and mucus clogging in cystic fibrosis (Thomas et al., 2014; Khan et al., 2019). A wide variety of studies have highlighted the proteolytic activity of elastase in causing structural changes, such as higher mean linear intercept and alveolar enlargement both in mice and in rats. Several changes resulted from elastase administration such as disorganized elastin, degradation of proteoglycans, and abnormal collagen remodeling. Regarding the dose and number of elastase challenges, many scientific groups demonstrated that mice subjected to five elastase administrations with a 1-week interval between them developed not only a more severe alveolar destruction, but also systemic manifestations, such as diaphragmatic dysfunction, weight loss, pulmonary arterial hypertension, and exercise intolerance (De et al., 2016; Crocetti et al., 2019; Giacalone et al., 2020). Thus, inhibition of elastase by chemical drugs has also been suggested as a way to recover from different inflammatory diseases. Unfortunately, the present anti-inflammatory drugs are only alleviating the symptoms of these diseases but not the progression of the disease (Barnes and Stockley, 2005). Sivelestat is the only approved drug working as a selective HNE inhibitor. However, clinical trials revealed an insufficient therapeutic efficacy of the drug in human severe lung injury and respiratory inflammation (Zeiher et al., 2004). Additionally, sivelestat has the risks of organ toxicity and poor pharmacokinetics (Tsai et al., 2015; Giacalone et al., 2020). The problem in developing new elastase inhibitors is the interference function of elastase with other proteases.

The recognition of neutrophil elastase as a promising target in chronic inflammatory diseases has increased (Wagner et al., 2016; Dittrich et al., 2018; Giacalone et al., 2020). This is why we have chosen to shed light on natural polypeptides as significant inhibitors of elastases, which could be used as hit-to-lead compounds for further development. This review basically summarizes the reported natural polypeptides of different sources (bacterial, plant, fungal, and animal origin) as potential inhibitors of elastase, and lists the relevant structures and activity relationship for the high active polypeptides-based inhibitors. Additionally, this review article offers information about the chemical structures and structural features of these compounds, structural elucidation including their absolute configuration, and structure-activity relationship.

Generally, natural peptides have poor absorption, distribution, metabolism, and excretion (ADME) properties with rapid clearance, sometimes low solubility, low permeability, and a short half-life. Strategies have been developed to improve peptide drugability through prolonging half-life, reducing renal clearance and proteolysis, and enhancing permeability. *In silico*, *in vitro*, and *in vivo* tools are available to evaluate ADME characteristics of natural peptides, and structural functional modification strategies are ongoing to improve peptide developability. Clear understanding and improving the physicochemical properties that govern peptide conformation is critical in assessing the impact on potency and ADME properties (*e.g.*, stability, permeability, and PK) (Di, 2014).

Porcine pancreatic elastase (PPE) has the advantage of being inexpensive and is able to induce features of lung damage and panacinar emphysema.

## Anti-elastase Polypeptides of Bacterial Origin

### Polypeptides From the Cyanobacteria *Lyngbya* spp.

**Figure 1:**
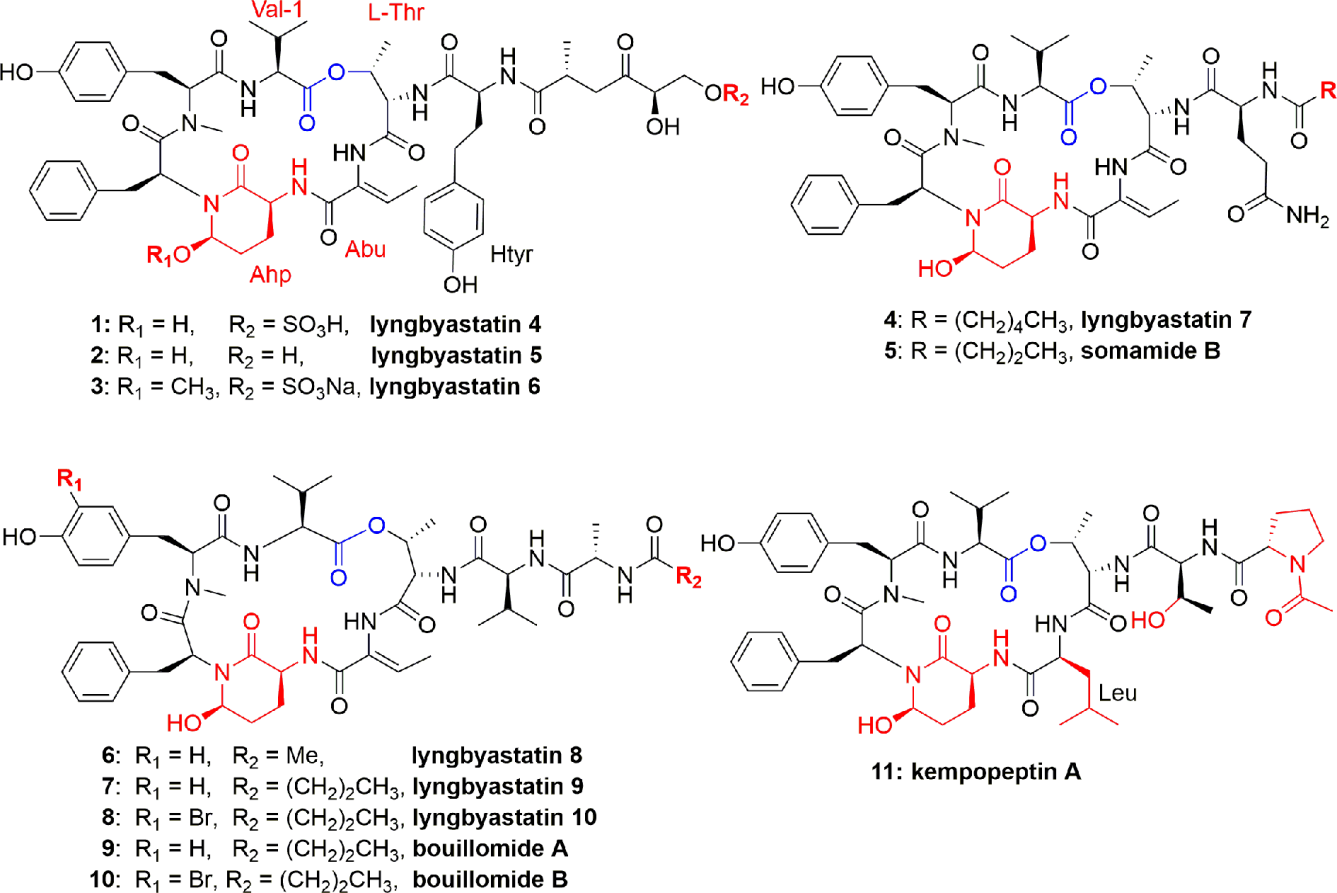
Structures of lynbyastatins, somamide B, Bouillomides, and kempopeptin A from different *Lyngbya* spp. They are cyclic depsipeptides containing Ahp unit; all (except kempopeptin A) having Abu unit neighbor to the Ahp unit. The Ahp residue contains a glutamate semi-aldehyde bonded as hemi-aminal to the amide nitrogen of the following aa; kempopeptin A is a new Ahp-containing depsipeptide and possessing N-acetylated Pro. The ester linkage for these depsipeptides was formed between a threonine OH group and the hydrophobic C-terminus of the precursor peptide.

The genus *Lyngbya* (marine cyanobacteria) is a prolific producer of peptides. The depsipeptide *lyngbyastatin* 4 (**1**) (**Figure 1**) is produced by the marine cyanobacterium *Lyngbya confervoides* from the Florida Atlantic coast. The planar structure of lyngbyastatin 4 (**1**) was confirmed by NMR measurements, whereas the absolute stereochemistry was corroborated through chiral HPLC analysis of its hydrolyzed moieties. Compound **1** contains an unusual aa homotyrosine and a residue of 3-amino-6-hydroxy-2-piperidone (Ahp). It selectively inhibited PPE *in vitro* with an IC_50_ = 0.03 µM (Matthew et al., 2007). In another study, compound **1** potently inhibited PPE and HNE (IC_50_ 0.041 ± 2.0 and 0.049 ± 1.4 µM, respectively) (Salvador et al., 2013). This compound showed no cytotoxicity to various cancerous cell lines. Lyngbyastatin 4 (**1**) is an analogue of several marine cyanobacterial compounds such as dolastatin 13, and it was revealed that many of the dolastatins are originated from cyanobacteria (Matthew et al., 2007).

Three more lyngbyastatins, 5-7 (**2-4**), and a cyclodepsipeptide somamide B (**5**) were discovered in the culture of cyanobacteria, *Lyngbya* spp., (South Florida). The chemical structures of compounds **2-5** (**Figure 1**) were established due to NMR measurements, while the absolute configuration was established based on Marfey's analysis of the acid hydrolysis. All these compounds **2-5** selectively and potently inhibited PPE (Taori et al., 2007). The potent PPE inhibitory activities of compounds **2-5** were observed with similar IC_50_ values of 0.0032 (**2**), 0.0033 (**3**), 0.0083 (**4**), and 0.0095 (**5**) µM (Taori et al., 2007). In another study, compound **4** potently inhibited PPE and HNE with IC_50_ values of 0.003 and 0.0023 µM, respectively (Salvador et al., 2013). Compound **4** potently and specifically inhibited elastase where it was tested at a single concentration of 10 µM against a group of 68 proteases and it showed preferential and complete inhibition for the serine proteases elastase, proteinase K, and chymotrypsin (Salvador et al., 2013). As part of the mode of action of these peptides, Salvador et al. studied their selective inhibitory activity against elastase through co-crystallization of the most potent inhibitor, lyngbyastatin 7 (**4**), with PPE. The co-crystal complex of the PPE−lyngbyastatin 7 was solved at 1.55 Å resolution. The study revealed that these compounds act as substrate mimics, where the Abu moiety (2-amino-2-butenoic acid) and the N-terminal residues occupy subsites S1 to S4. Besides a non-bonded interaction of ethylidene moiety of Abu unit with Ser203, it also binds to Gly201 and Ser222 through H-bonding, and an indirect H-bond with Thr44 through a molecule of water (Salvador et al., 2013). Due to the selectivity and potency of lyngbyastatin 7 (**4**) in inhibiting PPE, it was considered as a promising lead and subjected to further developmental studies (Luo and Luesch, 2020).

Marine cyanobacteria continue to furnish polypeptides, as *Lyngbya semiplena* (Tumon Bay, Guam) provided three cyclodepsipeptides, lyngbyastatins 8-10 (**6-8**) (**Figure 1**). These isolates were characterized by MS, ESIMS fragmentation, NMR, and chemical decomposition, and were found to have similar structural features to lyngbyastatin 4 (**1**). Compounds **6-8** inhibited PPE with IC_50_ 0.123, 0.21, and 0.12 µM, respectively (Kwan et al., 2009). Structure-activity relationship of compounds **6-8** was compared to that of lyngbyastatin 7 (**4**), and it was proposed that differences in structure of the side chain contributed to their potency reduction. In addition, the occurrence of hydrophobic moieties in the pendant chain is supposed to establish electrostatic and H-bonding interactions with the enzyme. However, the presence of a bromide atom didn`t significantly impact the activity of **8** compared to those of **6** and **7** (Kwan et al., 2009).

The two depsipeptide analogues of dolastatin 13, bouillomides A (**9**) and B (**10**) (**Figure 1**), were characterized and found in the extract of the cyanobacterium *Lyngbya bouillonii*. Both compounds selectively inhibited PPE with the same IC_50_ value of 1.9 µM (Rubio et al., 2010). Taori et al. (2008) identified a cyclodepsipeptide named kempopeptin A (**11**) (**Figure 1**) which was isolated from *Lyngbya* spp. This compound exhibited an IC_50_ against PPE of 0.3 μM. Peptide **11** selectively inhibited elastase activity by binding through the aa residue between Ahp and Thr (Taori et al., 2008).

As a conclusion, in compounds **1**-**11**, a 6-unit cyclic core, having Ahp and a pendant side chain, is very rigid, due to H bonding between Ahp and Val, which makes the hydrolysis of the inhibitor difficult. The Abu moiety adjacent to the Ahp unit makes the elastase more susceptible to the lyngbyastatin series (Kwan et al., 2009) due to H-bonding.

**Figure 2:**
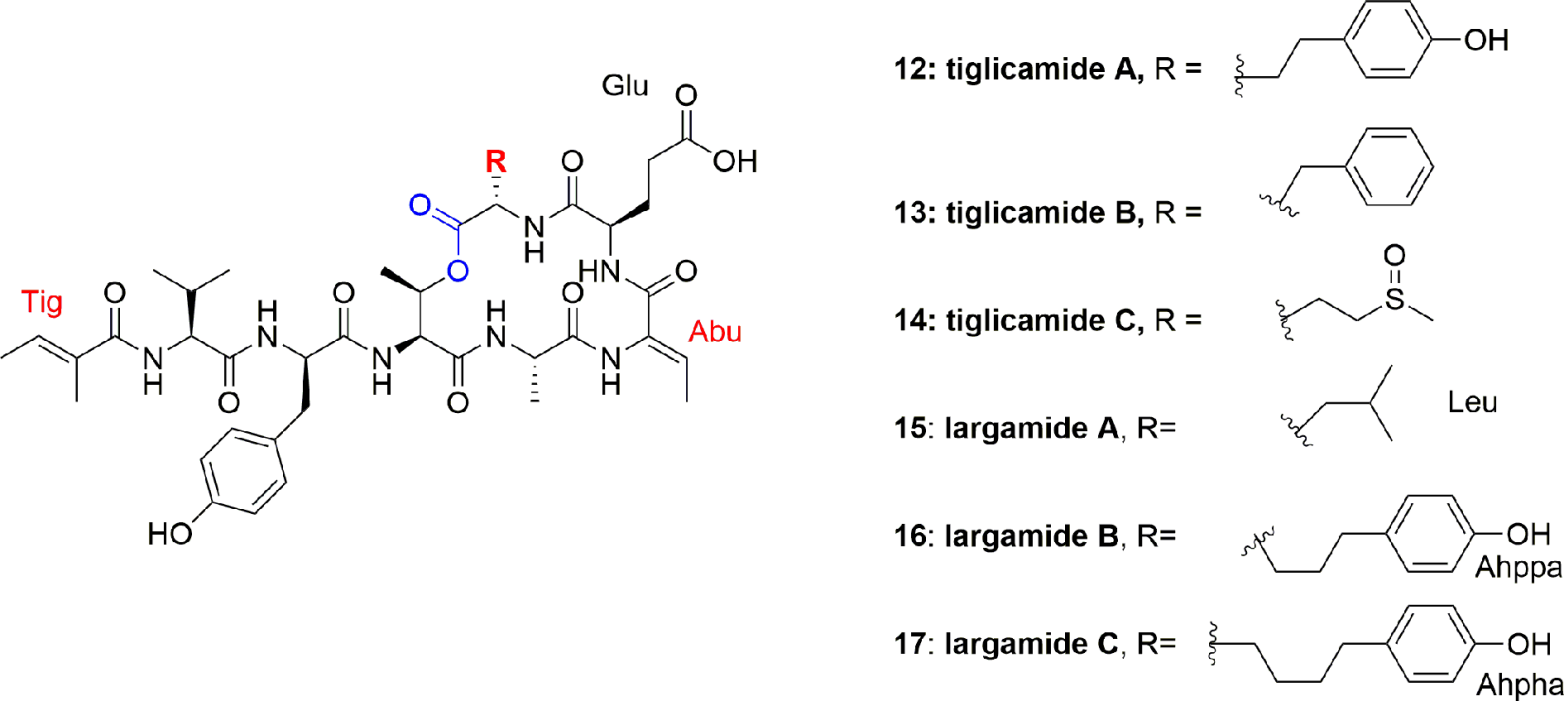
Structures of tiglicamides A-C (**12**-**14**) and largamides A-C (**15**-**17**) from *Lyngbya confervoides*, all are cyclic depsipeptides with unusual tiglic acid and Abu moieties and without Ahp unit.

*Lyngbya confervoides* (southeastern Florida) produced more cyclodepsipeptides, tiglicamides A-C (**12-14**), and largamides A-C (**15-17**) (**Figure 2**). The unique feature of these compounds is that they contain an unusual moiety, viz. tiglic acid. Compounds **12-14** moderately inhibited PPE *in vitro* with IC_50_ between 2.1 and 7.3 µM (Matthew et al., 2009b). Authors further reported 2-3 times lower activity, against PPE or other mammalian elastases, of compounds **12-14** as compared to lyngbyastatins 5-7 (**2-4**) (Matthew et al., 2007; Taori et al., 2007). The investigators also identified the corresponding largamide methyl esters of compounds **12–14**, which were supposed to be the isolation artifacts, however, these ester derivatives helped to establish SAR, and it was proposed that carboxylic acid moiety is not responsible for the activity of elastase inhibition, since methylation at that position did not affect the activity (Matthew et al., 2009b). The largamides A-C (**15-17**) also have medium inhibition of PPE activity *in vitro* with IC_50_ between 0.5 and 1.4 μM but no activity against chymotrypsin and trypsin up to 50 μM. It was suggested that the carboxylic acid functionality of the glutamic acid moiety in largamides A-C (**15-17**) play a role in elastase inhibition. They showed moderate *in vitro* activity against PPE in a dose-dependent manner with IC_50_ 1.4, 0.5, and 1.2 μM, respectively but remain inactive against the tested cancerous cell lines IMR-32, HT29, and U2OS (Matthew et al., 2009a).

### Polypeptides From the Cyanobacterium *Symploca* sp.

**Figure 3:**
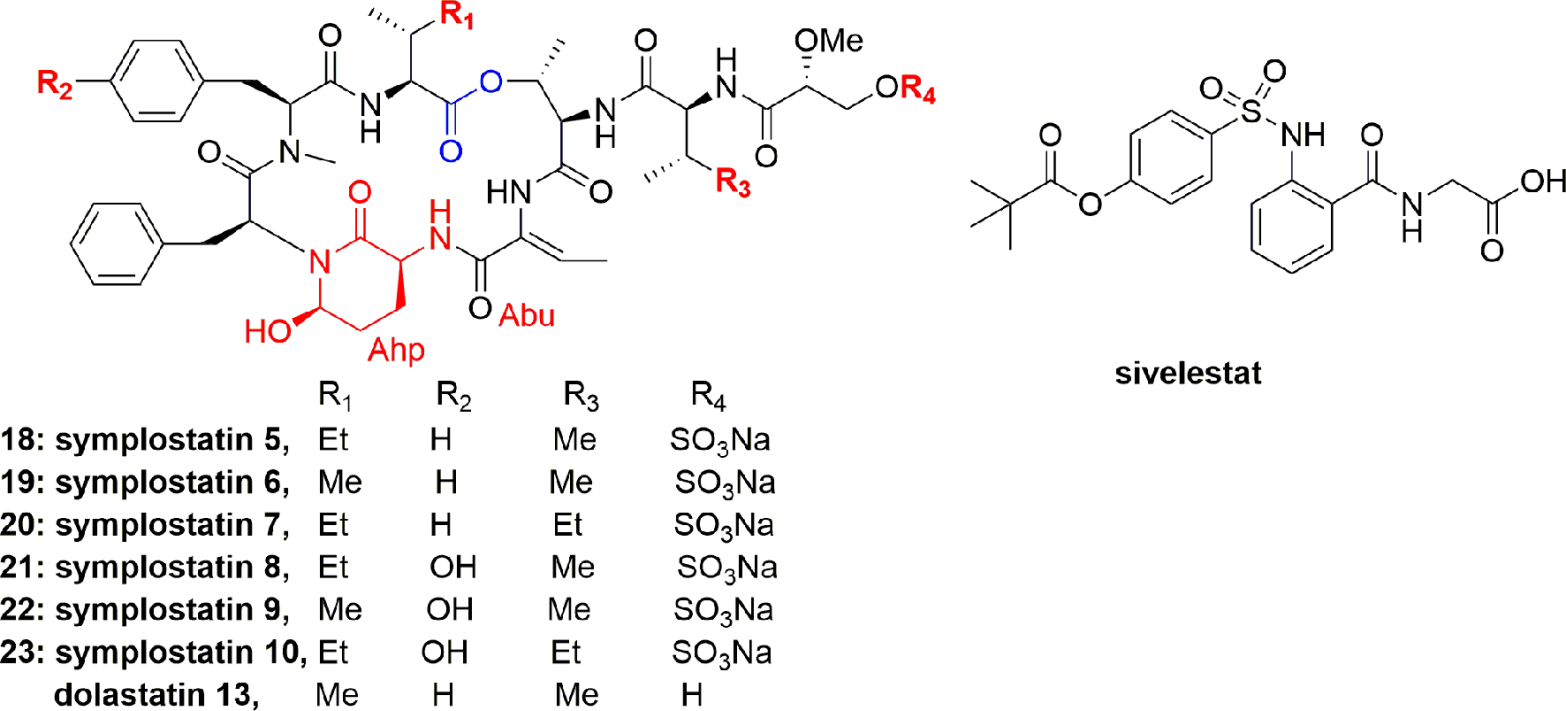
Structures of symplostatins 5-10 (**18**-**23**) isolated from cyanobacterium *Symploca* sp. and sivelestat. They comprise a 19-membered ring containing one lactone, 5 lactam links, and having Ahp and Abu units.

Salvador et al. isolated the symplostatins 5-10 (**18-23**) (**Figure 3**) from the red cyanobacterium *Symploca* sp. (Cetti Bay, Guam). Compounds **21**-**23** potently inhibited PPE with IC_50_ values 0.043, 0.037, and 0.044 µM, respectively, and also potently inhibited HNE with IC_50_ values of 0.041, 0.028, and 0.021 µM, respectively, and compounds **18**-**20** gave higher IC_50_ values from 0.121 to 0.195 µM. Through SAR study and X-ray co-crystal structural analyses, it was analyzed that compounds **21-23**, containing N-Me-Tyr, have a higher potential than the N-Me-Phe analogues **18-20** in elastase inhibition. It is further observed that Ile to Val substitution and pendant side chain have no effect on the activity. Compounds **21-23** and lyngbyastatins 4 (**1**) and 7 (**4**) exhibited more potential in inhibiting the PPE than the standard drug sivelestat, whereas compounds **18-20** exhibited higher IC_50_ values comparable to the activity of sivelestat (Salvador et al., 2013).

To study the cytoprotective effects of **18** against elastase-induced antiproliferation and apoptosis, investigators utilized the BEAS-2B (bronchial epithelial cell line), an SV-40. Compound **18** prevented the antiproliferative effect of elastase on MTT assayagainst BEAS-2B cell with an IC_50_ 0.077 µM at 24 h (Salvador et al., 2013). A striking feature of compound **18** is that it showed a comparable potential to sivelestat and didn`t show any toxicity on the bronchial cells. In conclusion, it was stated that symplostatin 5 (**18**) alleviated chronic pulmonary diseases. Therefore, compound **18** offers a remarkable window to establish the molecular basis and biomarkers for elastase inhibitors that can aid in the creation of second generation inhibitors (Salvador et al., 2013).

### Polypeptide From the Cyanobacterium *Stigonema* sp.

**Figure 4:**
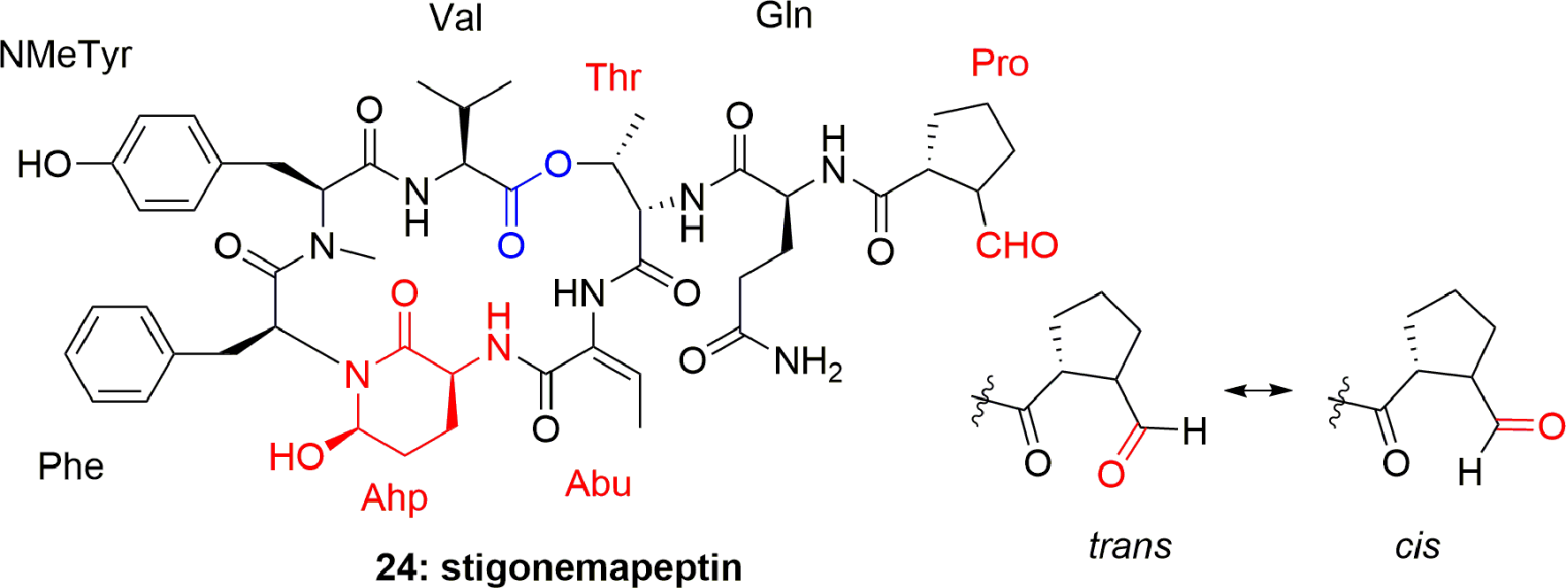
Structure of stigonemapeptin (**24**) isolated from the freshwater cyanobacterium *Stigonema* sp. (northern Wisconsin).

Another depsipeptide, stigonemapeptin (**24**) (**Figure 4**), was produced by *Stigonema* sp. (a freshwater cyanobacterium from northern Wisconsin). The characteristic feature of **24** was that it contains an Ahp residue and the modified aa Abu and N-formylated proline residue. Compound **24** showed *in vitro* PPE inhibition with IC_50_ 0.3 μM (Kang et al., 2012).

### Polypeptides From the Cyanobacteria *Oscillatoria* spp.

**Figure 5:**
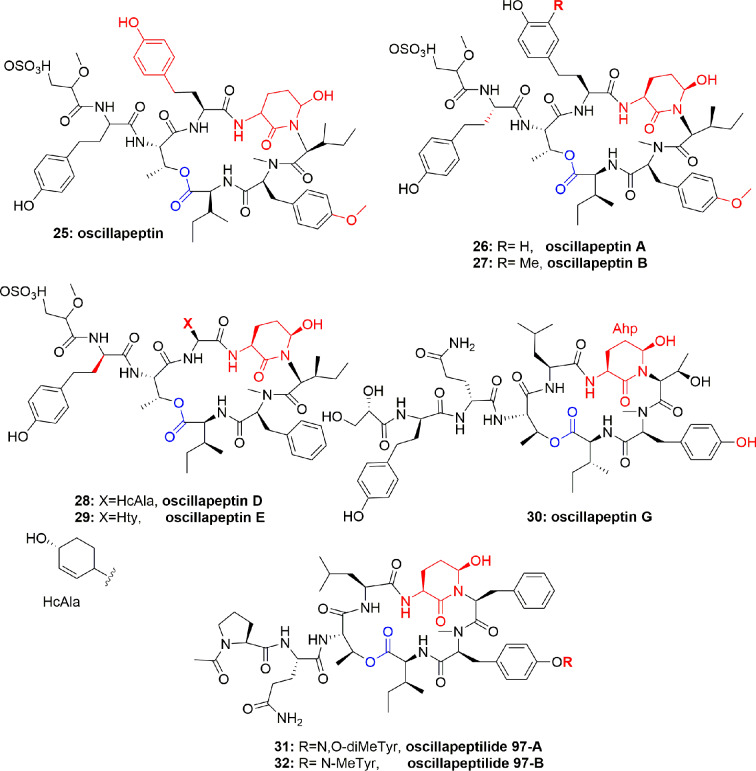
Structures of scyptolins A and B (**58–59**); they hold a 19-membered ring containing one lactone (involving the side chain hydroxyl of a threonine residue), an Ahp residue and 5 lactam links.

The cyclic depsipeptide oscillapeptin (**25**) (**Figure 5**) was obtained from another cyanobacterium, *Oscillatoria agardhii* (NIES-204). Compound **25** inhibited elastase enzyme activity with IC_50_ 2.5 µM. This depsipeptide **25** contains Ahp, an acyl group, and 7 aa or their derivatives and hence it is related to dolastatin 13 and micropeptins. The unusual feature of oscillapeptin (**25**) is that it has two homotyrosine units and N,O-dimethyltyrosine moiety, which is rare in natural peptides. This unique feature of similar peptides with a variety of aa compositions offers interest to biochemists to study their biosynthetic pathway (Shin et al., 1995). During other investigations, *Oscillatoria agardhii* in three different cultured conditions produced oscillapeptins A, B, D, and E (**26-29**) (**Figure 5**). These compounds inhibited elastase with IC_50_ 2.5, 4.2, 2.6, and 2.7 µM, respectively (Itou et al., 1999).

**Figure 6:**
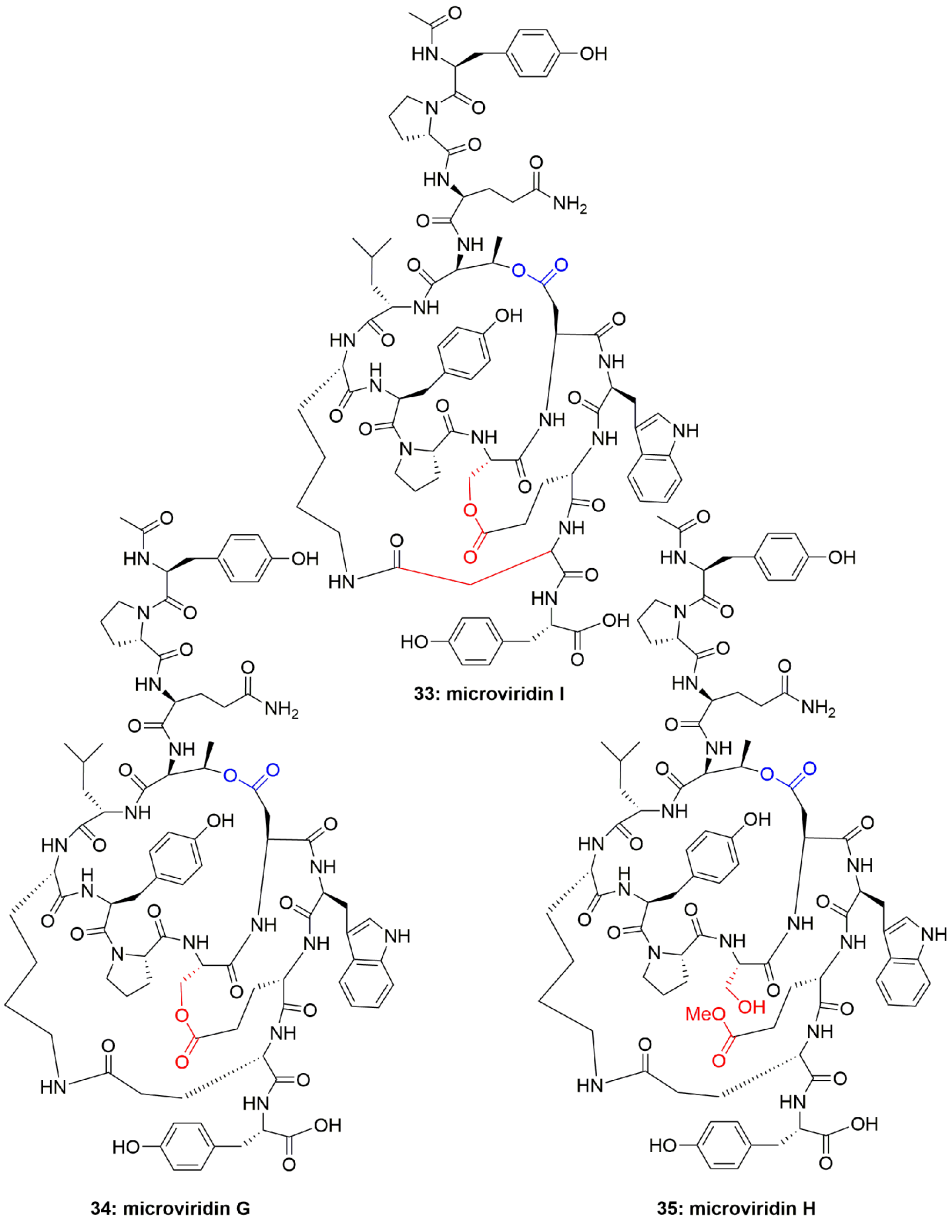
Structures of microviridins I, G, and H.

The cyclic depsipeptides oscillapeptin G (**30**) and oscillapeptilides 97-A (**31**) and -B (**32**) (**Figure 5**) were produced by the toxic strains of *Oscillatoria agardhii*, having the elastase inhibition property with IC_50_ 1.0, 6.9, and 3.9 µM, respectively (Fujii et al., 2000). The tricyclic peptide microviridin I (**33**) (**Figure 6**) was obtained from the non-toxic strain of *Oscillatoria agardhii*. This compound showed inhibitory activity towards elastase release with IC_50_ 1.9 µM (Fujii et al., 2000).

### Polypeptides From the Cyanobacteria *Nostoc* spp.

Another cyanobacterium, *Nostoc minutum* (NIES-26), inhabiting fresh water, has been reported to produce microviridin-type peptides, which were identified as microviridins G (**34**) and H (**35**) (**Figure 6**). Both compounds potently inhibited elastase activity with IC_50_ 1.0 and 1.7 µM, respectively (Murakami et al., 1997). Although investigators did not comment on the structural features and level of activity of **34** and **35**, this may be because both the compounds are highly active, however, **34** is more active than **35** which could be attributed to additional cyclization in the form of lactone in compound **34**, that results in slightly increased hydrophobicity.

**Figure 7:**
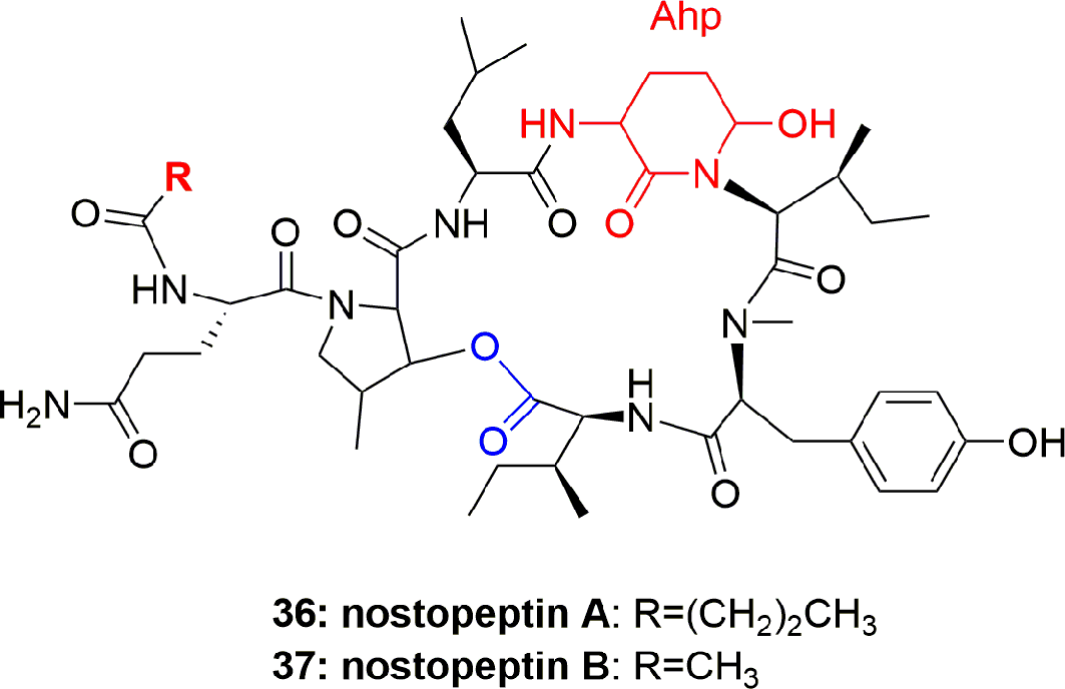
Structures of nostopeptins A and B.

Nostopeptins A (**36**) and B (**37**) (**Figure 7**) were produced by the cyanobacterium *Nostoc Minutum* (NIES-26). These compounds were characterized as cyclic depsipeptides-containing Ahp but not Abu unit. Both the compounds inhibited elastase with IC_50_ 1.3 and 1.2 µM, respectively (Okino et al., 1997).

**Figure 8:**
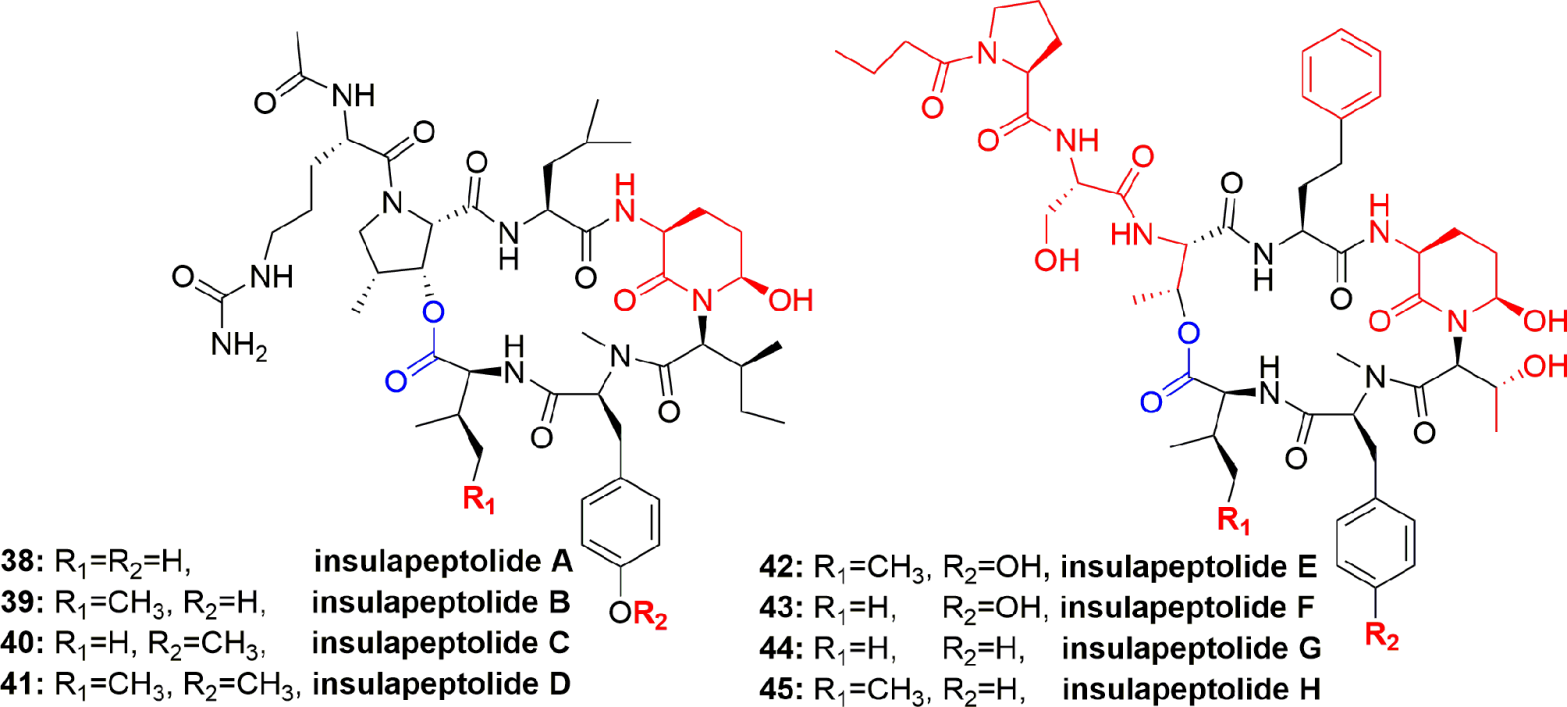
Structures of insulapeptolides A–H (**38**-**45**), all having Ahp unit but not Abu unit. These depsipeptides contain a 19-membered ring created from six aa residues by the formation of lactone between a side chain OH group of the first residue and the C-terminal carboxylate.

From the cyanobacterium *Nostoc insulare* (Nostocales), eight new cyanopeptolins named insulapeptolides A–H (**38-45**) (**Figure 8**) were isolated guided by their bioactivity toward the target enzyme HLE, MALDITOF, and molecular biological analysis. The insulapeptolides A–H (**38-45**) selectively inhibited HLE with IC_50_ 0.1, 0.1, 0.09, 0.08, 3.2, 1.6, 3.5, and 2.7 µM, respectively (Mehner et al., 2008).

### Polypeptides From the Cyanobacteria *Microcystis* spp.

**Figure 9:**
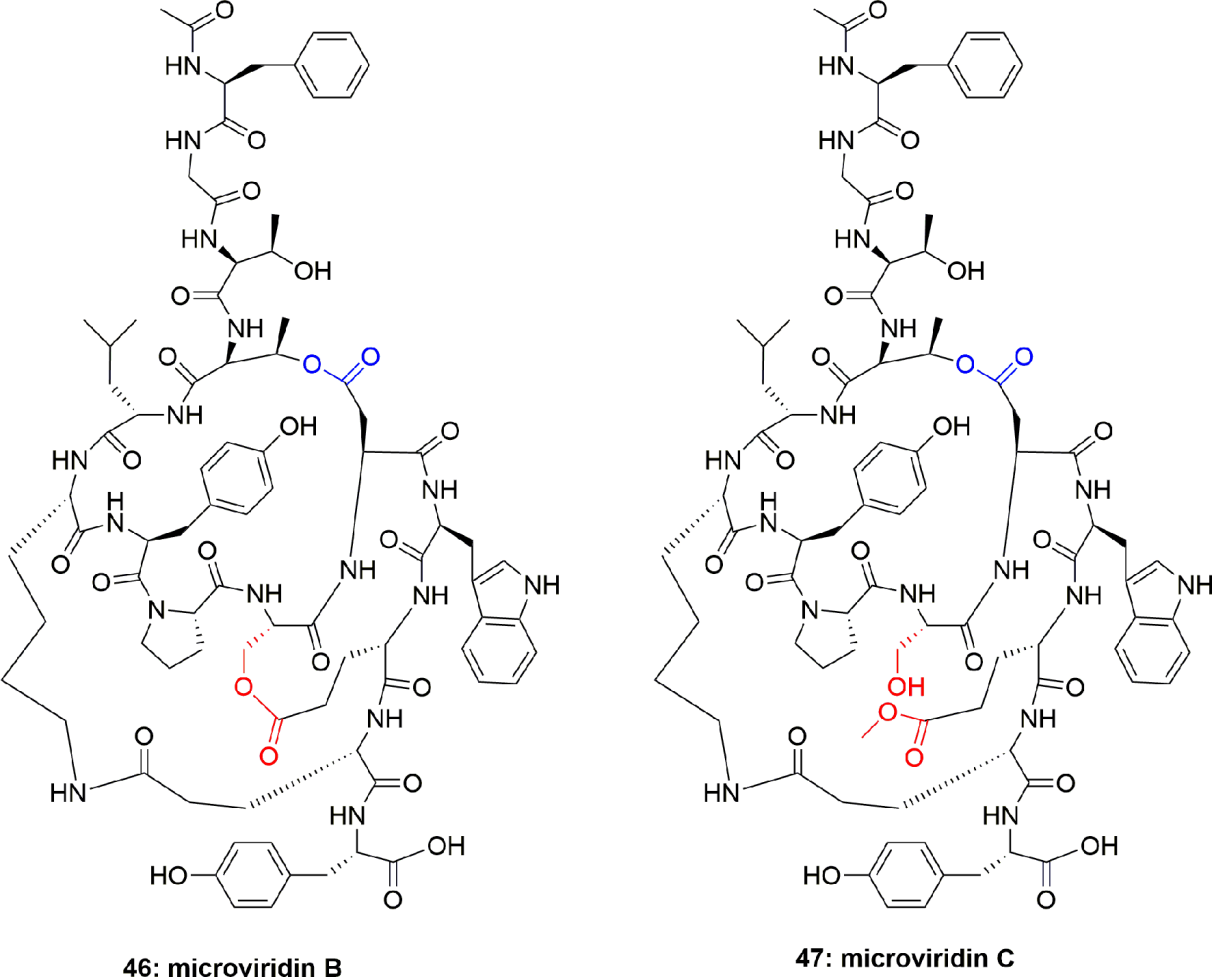
Structures of microviridins B and C isolated from *Microcystis aeruginosa*.

The new microviridins B (**46**) and C (**47**) (**Figure 9**) were also obtained from *Microcystis aeruginosa*. These compounds potentially inhibited elastase activity with IC_50_ 2.6 and 4.8 µM, respectively (Okino et al., 1995).

**Figure 10:**
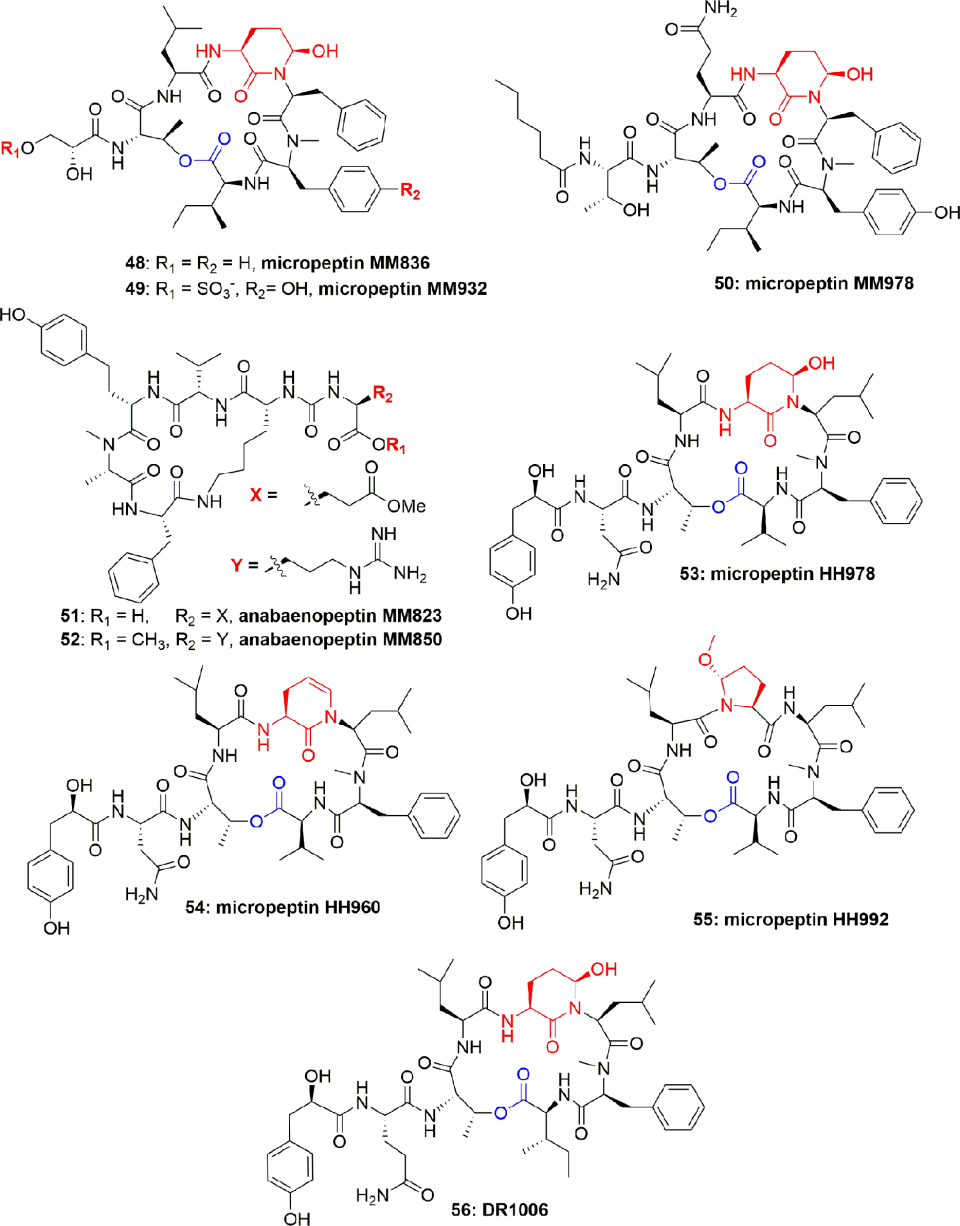
Structures of micropeptins **48-50** are with Ahp unit, **53**-**56** are also with Ahp unit, and the anabaenopeptins **51-52** are without Ahp unit. These micropeptins are depsipeptides. The anabaenopeptins are hexapeptides and cyclic through the cyclization of the C-terminal aa carboxyl to the ƹ-amine residue of the N-terminal-D-lysine, while the α-amine of this lysine fraction is linked through an ureido-bridge to the side chain of another aa. All anabaenopeptins described to date from cyanobacteria, contain D-lysine, and have an L-absolute configuration for the other aa.

The aqueous extract of *Microcystis* sp. produced several polypeptides including micropeptins MM836 (**48**), MM932 (**49**), MM978 (**50**), anabaenopeptins MM823 (**51**), and MM850 (**52**) (**Figure 10**). These metabolites were purified using sephadex LH-20 and RP-ODS column. These compounds inhibited the elastase activity with IC_50_ between 4.4 and 50.0 µM, with compound **49** displaying the most potential (IC_50_ = 4.4 µM) (Zafrir-ilan and Carmeli, 2010) (**Table 1**). No Data is available on either mode of action or the cytotoxicity of compounds **48**-**52**. However, comparison of the IC_50_ values disclosed that the degree of binding of the micropeptins to the elastase catalytic pocket increases when residue-2 is comprised of tyrosine instead of phenylalanine. Anabaenopeptins are mild inhibitors of serine proteases as compared to the micropeptins, however, among anabaenopeptins, the higher binding affinity of **52** is attributed to an OMeArg in the ureido-bridge of **52**, which is OMeGlu in **51**.

**Table 1:** Natural polypeptides having activity against elastase and their IC_50s_.

	Compound	Name	IC_50_ against HNE/µM	IC_50_ against PPE/µM	Reference
Anti-elastase polypeptides of bacterial origin	**1**	Lyngbyastatin 4	0.049	0.03	(Matthew et al., 2007; Salvador et al., 2013)
				In another study, 0.04	
	**2**	Lyngbyastatin 5		0.0032	(Taori et al., 2007; Salvador et al., 2013)
	**3**	Lyngbyastatin 6		0.0033	(Taori et al., 2007; Salvador et al., 2013)
	**4**	Lyngbyastatin 7	0.0023	0.0083	(Taori et al., 2007; Salvador et al., 2013)
				In another study, 0.003	
	**5**	Somamide B	0.0095	0.0095	(Taori et al., 2007; Salvador et al., 2013)
	**6**	Lyngbyastatin 8		0.123	(Kwan et al., 2009)
	**7**	Lyngbyastatin 9		0.210	(Kwan et al., 2009)
	**8**	Lyngbyastatin 10		0.120	(Kwan et al., 2009)
	**9**	Bouillomide A		1.9	(Rubio et al., 2010)
	**10**	Bouillomide B		1.9	(Rubio et al., 2010)
	**11**	Kempopeptin A		0.3	(Taori et al., 2008)
	**12**	Tiglicamide A		2.1	(Matthew et al., 2009b)
	**13**	Tiglicamide B		6.9	(Matthew et al., 2009b)
	**14**	Tiglicamide C		7.3	(Matthew et al., 2009b)
	**15**	Largamide A		1.4	(Matthew et al., 2009a)
	**16**	Largamide B		0.5	(Matthew et al., 2009a)
	**17**	Largamide C		1.2	(Matthew et al., 2009a)
	**18**	Symplostatin 5		0.068	(Salvador et al., 2013)
	**19**	Symplostatin 6		0.089	(Salvador et al., 2013)
	**20**	Symplostatin 7		0.077	(Salvador et al., 2013)
	**21**	Symplostatin 8	0.041	0.043	(Salvador et al., 2013)
	**22**	Symplostatin 9	0.028	0.037	(Salvador et al., 2013)
	**23**	Symplostatin 10	0.021	0.044	(Salvador et al., 2013)
	**24**	Stigonemapeptin		0.3	(Kang et al., 2012)
	**25**	Oscillapeptin		2.5	(Shin et al., 1995)
	**26**	Oscillapeptin A		2.5	(Itou et al., 1999)
	**27**	Oscillapeptin B		4.2	(Itou et al., 1999)
	**28**	Oscillapeptin D		2.6	(Itou et al., 1999)
	**29**	Oscillapeptin E		2.7	(Itou et al., 1999)
	**30**	Oscillapeptin G		1.0	(Fujii et al., 2000)
	**31**	Oscillapeptilide 97-A		6.9	(Fujii et al., 2000)
	**32**	Oscillapeptilide 97-B		3.9	(Fujii et al., 2000)
	**33**	Microviridin I		1.9	(Fujii et al., 2000)
	**34**	Microviridin G		1.0	(Murakami et al., 1997)
	**35**	Microviridin H		1.7	(Murakami et al., 1997)
	**36**	Nostopeptin A		1.3	(Okino et al., 1997)
	**37**	Nostopeptin B	1.2		(Okino et al., 1997)
	**38**	Insulapeptolide A	0.1		(Mehner et al., 2008)
	**39**	Insulapeptolide B	0.1		(Mehner et al., 2008)
	**40**	Insulapeptolide C	0.09		(Mehner et al., 2008)
	**41**	Insulapeptolide D	0.08		(Mehner et al., 2008)
	**42**	Insulapeptolide E	3.2		(Mehner et al., 2008)
	**43**	Insulapeptolide F	1.6		(Mehner et al., 2008)
	**44**	Insulapeptolide G	3.5		(Mehner et al., 2008)
	**45**	Insulapeptolide H	2.7		(Mehner et al., 2008)
	**46**	Microviridin C		2.6	(Okino et al., 1995)
	**47**	Microviridin C		4.8	(Okino et al., 1995)
	**48**	Micropeptin MM836		45.5	(Zafrir-ilan and Carmeli, 2010)
	**49**	Micropeptin MM932		4.4	(Zafrir-ilan and Carmeli, 2010)
	**50**	Micropeptin MM978		19.1	(Zafrir-ilan and Carmeli, 2010)
	**51**	Anabaenopeptin MM823		50.5	(Zafrir-ilan and Carmeli, 2010)
	**52**	anabaenopeptin MM850		14.3	(Zafrir-ilan and Carmeli, 2010)
	**53**	Micropeptin HH978		17.6	(Lodin-Friedman and Carmeli, 2013)
	**54**	Micropeptin HH960		55.5	(Lodin-Friedman and Carmeli, 2013)
	**55**	Micropeptin HH992		16.9	(Lodin-Friedman and Carmeli, 2013)
	**56**	micropeptin DR1006		13.0	(Adiv et al., 2010)
	**57**	Molassamide		0.03	(Gunasekera et al., 2010)
	**58**	Scyptolin A		1.6	(Matern et al., 2001; Matern et al., 2003a)
	**59**	Scyptolin B		1.4	(Matern et al., 2001; Matern et al., 2003a)
	**60**	planktopeptin BL1125		0.096	(Grach-pogrebinsky et al., 2003)
	**61**	planktopeptin BL843		1.7	(Grach-pogrebinsky et al., 2003)
	**62**	planktopeptin BL1061		0.040	(Grach-pogrebinsky et al., 2003)
	**63**	brunsvicamide A	3.1		(Sisay et al., 2009)
	**64**	Brunsvicamide B	2.0		(Sisay et al., 2009)
	**65**	Brunsvicamide C	4.4		(Sisay et al., 2009)
	**66**	FR901277	0.2	0.3	(Nakanishi et al., 2000b)
	**67**	FR134043	0.04		(Nakanishi et al., 2000a)
	**68**	YM-47141	1.5		(Orita et al., 1995; Yasumuro et al., 1995)
	**69**	YM-47142	3.0		(Orita et al., 1995; Yasumuro et al., 1995)
Anti-elastase polypeptide of plant origin	**70**	Ixorapeptide II		5.6	(Lee et al., 2010)
Anti-elastase polypeptides of fungal origin	**71**	Desmethylisaridin C2	10.0		(Chung et al., 2013)
	**72**	Isaridin E	12.7		(Chung et al., 2013)
	**73**	Isaridin C2	12.1		(Chung et al., 2013)
	**74**	Roseocardin	15.1		(Chung et al., 2013)

*Microcystis aeruginosa* (IL-399) synthesized micropeptins HH978 (**53**), HH960 (**54**), and HH992 (**55**) (**Figure 10**). Compounds **53-55** inhibited elastase with IC_50_ 17.6, 55.5, and 16.9 μM, respectively, but not thrombin and trypsin at a concentration of 45.5 μM, which showed their selectivity towards elastase enzyme. Comparison of the elastase inhibitory potential of **53** and **54** revealed the importance of the Ahp-6-OH group. However, in contrast, the comparison of the anti-elastase potential of compounds **53** and **55** revealed that the enzyme is liberal to the structural variation and also accepts the Mpc moiety (Lodin-Friedman and Carmeli, 2013). Another micropeptin, DR1006 (**56**) (**Figure 10**), has also been reported as a metabolite of *Microcystis aeruginosa* and inhibited elastase with an IC_50_ 13.0 µM (Adiv et al., 2010). The SAR studies with other co-isolated compounds concluded that the elastase inhibitory activity of compound **56** can be attributed to the leucine moiety in the 5^th^ position from the C-terminus. This conclusion is also consistent with the activity of other similar compounds (Adiv et al., 2010).

### Polypeptide From the Cyanobacterium *Dichothrix utahensis*

**Figure 11:**
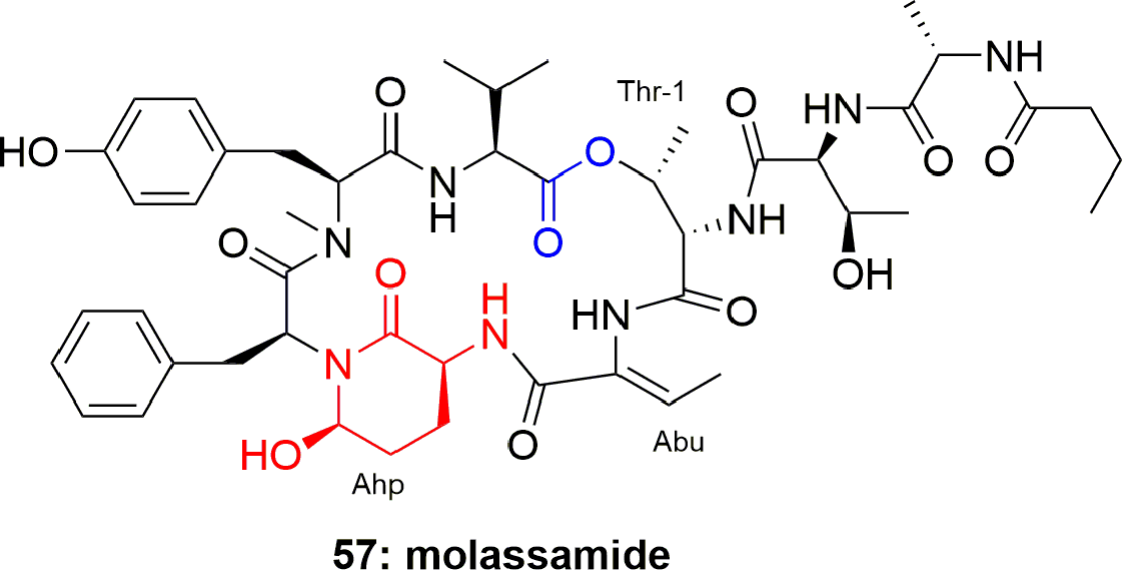
Structure of molassamide (**57**) has both Ahp and Abu units.

Molassamide (**57**) (**Figure 11**), an analogue of dolastatin, is considered to be the first peptide separated from marine cyanobacterial assemblages of *Dichothrix utahensis* (from Brewer's Bay, Virgin Islands and from the Molasses Reef, Florida). The structure of **57** was established through NMR spectroscopic techniques, while the absolute configuration at chiral centers was assigned through chiral HPLC analysis of the hydrolyzed products. Compound **57** exhibited potent protease-inhibition activity, with IC_50_ 0.03 and 0.23 µM against PPE and chymotrypsin, respectively (Gunasekera et al., 2010).

### Polypeptides From the Cyanobacterium *Scytonema hofmanni*

**Figure 12:**
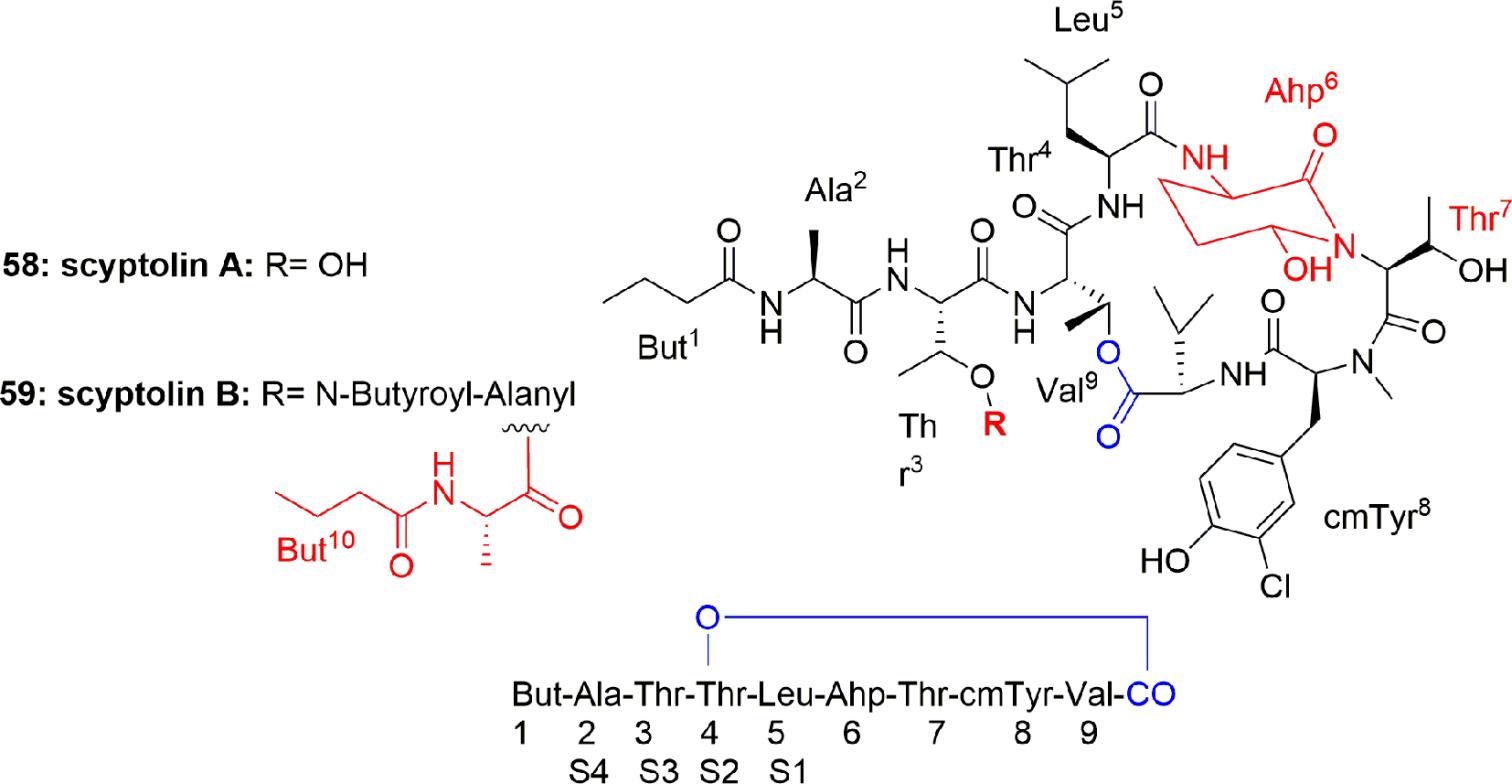
Scyptolins A and B (**58**-**59**) hold a 19-membered ring containing one lactone (involving the side chain hydroxyl of a threonine residue), an Ahp residue and 5 lactam links.

Another two cyclic depsipeptides, scyptolins A (**58**) and B (**59**) (**Figure 12**), were produced by *Scytonema hofmanni* PCC 7110. Spectroscopic based analysis of their structures revealed that these metabolites have a unique side chain and an uncommon moiety 3′-chloro-*N*-methyl-Tyr. Compounds **58** and **59** consisted of the N-acylated peptide But^1^-Ala^2^-Thr^3^-Thr^4^-Leu^5^-Ahp^6^-Thr^7^-cmTyr^8^-Val^9^, to build a 19-membered ring through esterifying the Val^9^ COOH with the Thr^4^ OH. It is further explained that OH of the Thr^3^ residue in compound **59** has another esterification bond with the N-butyroyl-Ala. Both the compounds **58** and **59** are reported to possess selective inhibition of PPE *in vitro* with IC_50_ 1.6 and 1.4 µM, respectively (Matern et al., 2001; Matern et al., 2003a). The crystal structure of scyptolin A-PPE demonstrated that the elastase inhibitor occupies the prominent subsites S1 through S4 of the enzyme, and this rigid structure banned hydrolysis of the complex (Matern et al., 2003b). The above studies suggest that the type of aa present in between Thr and Ahp define the selective inhibition of serine proteases, which is attributed to the preferences of the different binding to specific pockets of the enzyme (Matern et al., 2003b). The scyptolins` selectivity of elastase is likely regulated by the moiety in position 5, which corresponds to the P1 position of a substrate, which is leucine in the scyptolins (McDonough and Schofield, 2003).

### Polypeptides From the Cyanobacterium *Planktothrix rubescens*

**Figure 13:**
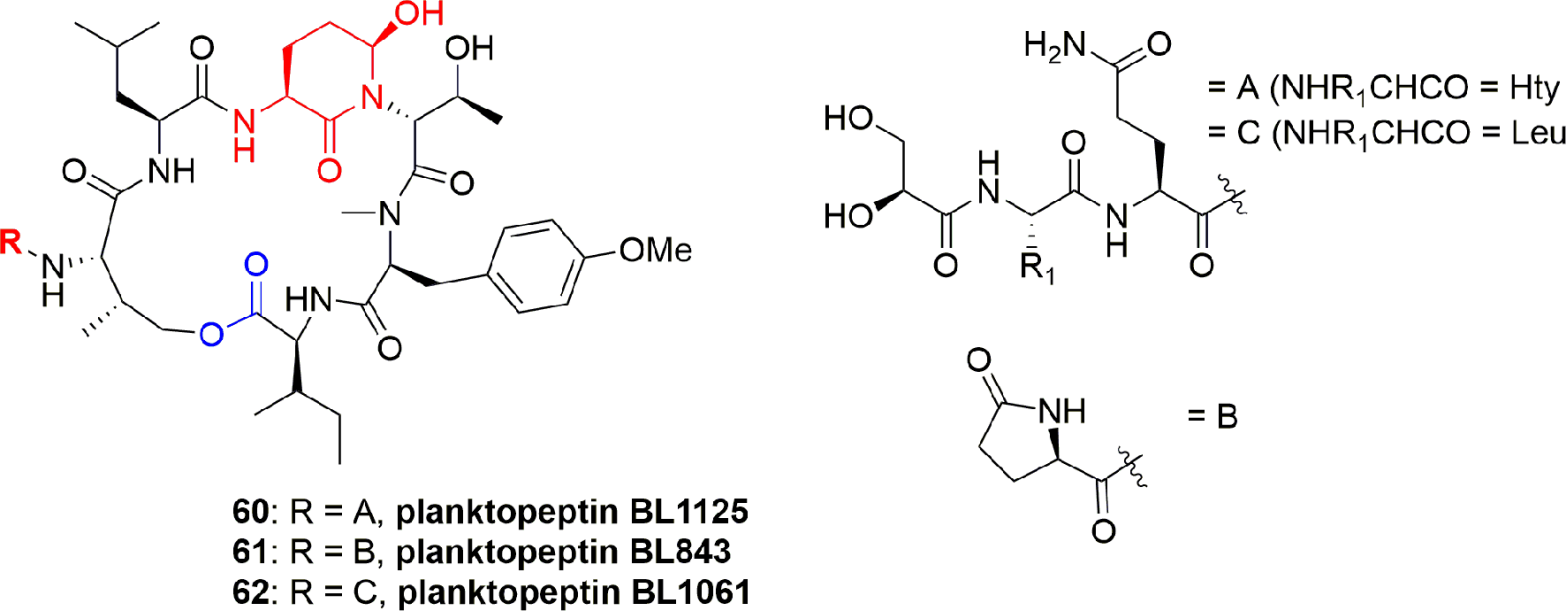
Structures of planktopeptins BL1125 (**60**), BL843 (**61**), and BL1061 (**62**) separated from *Planktothrix rubescens*.

Three planktopeptins, BL1125 (**60**), BL843 (**61**), and BL1061 (**62**) (**Figure 13**), were separated from the cyanobacterium *Planktothrix rubescens*. The three compounds **60-62** inhibited the activity of elastase enzyme with IC_50_ 0.096, 1.7, and 0.040 µM, respectively. It is stated that the flexible side chain moiety of compounds **60** and **62** is the factor for selectivity of elastase enzyme when compared with the activity for other enzymes (Grach-pogrebinsky et al., 2003).

### Polypeptides of the Cyanobacterium *Tychonema* sp.

**Figure 14:**
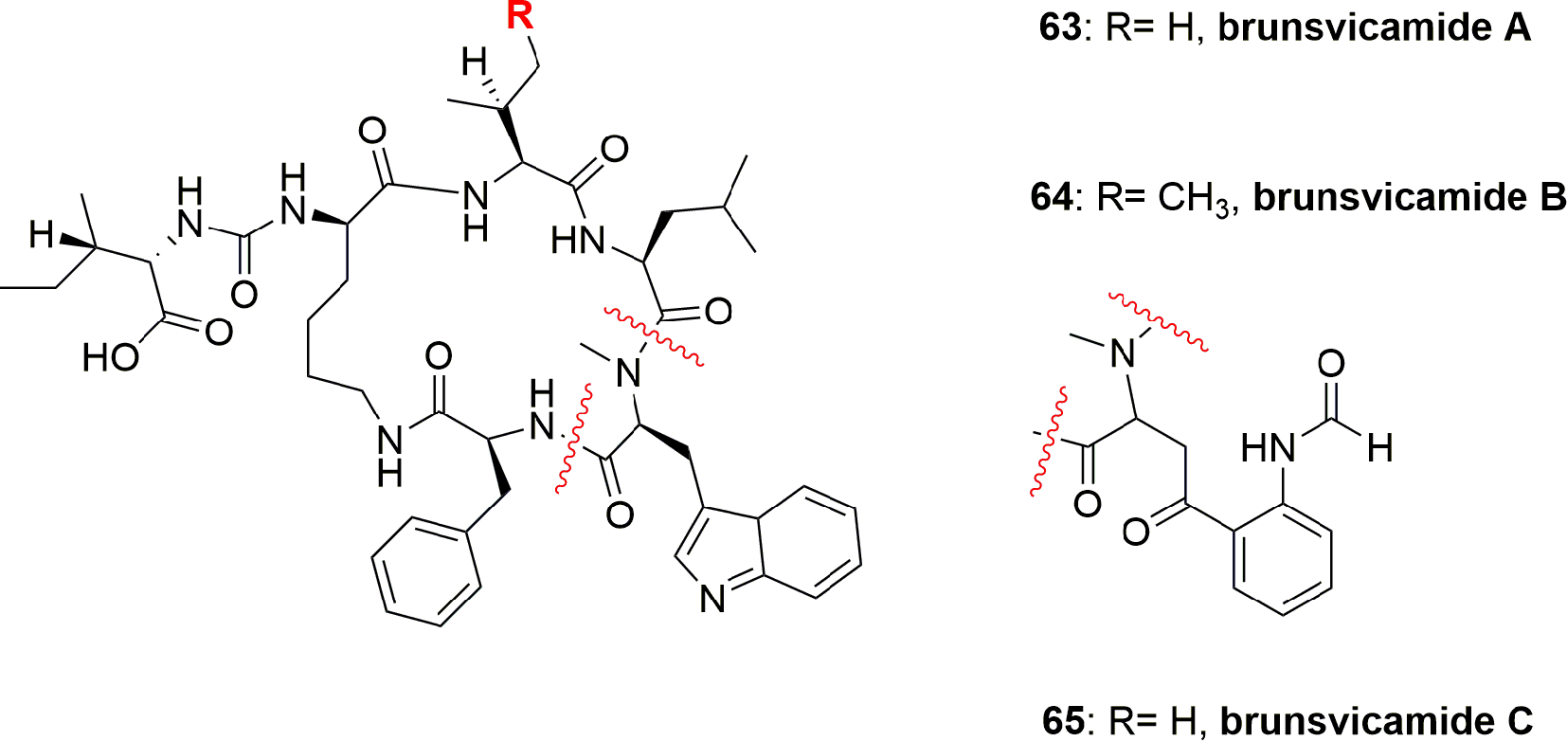
Structures of brunsvicamides A-C (**63**-**65**) obtained from *Tychonema* sp.

The brunsvicamides A-C (**63**-**65**) (**Figure 14**) obtained from the cyanobacterium *Tychonema* sp. were found to comprise six aa: five aa constitute a 19-membered ring skeleton, while the sixth aa is attached through urea moiety to the α-amino moiety of the D-Lys. These compounds have a structural similarity to those of anabaenopeptins, which have a D-Lys-urea moiety linked to a terminal aa and an N-methylated peptide bond in common, which differ in their aa sequence. Compounds **63**-**65** exhibit high selectivity for HLE inhibition with Ki 1.1, 0.70, and 1.6 µM, respectively, and having IC_50_ 3.1, 2.0, and 4.4 µM, respectively. The mode of action of these compounds has been investigated through modeling with the most active metabolite, brunsvicamide B (**64**). A reference co-crystal structure of scyptolin A and PPE was used, since the cyclic peptide core of scyptolin A has exactly the same number of aa and atoms as brunsvicamides. Based on the structural similarity, the investigators studied if the cyclic core of scyptolin A mimics the brunsvicamides A-C. Thus, the computational modeling suggested that brunsvicamides A–C might act by an inhibition mechanism which is similar to scyptolin A. The crystallographic data of the complex structure scyptolin A– elastase revealed that the active site of elastase was occupied by the macrocycle of scyptolin A in such a way as to prevent the access of water to make the cleavage difficult (Sisay et al., 2009).

### Polypeptides of the Bacterium *Streptomyces resistomicificus*

**Figure 15:**
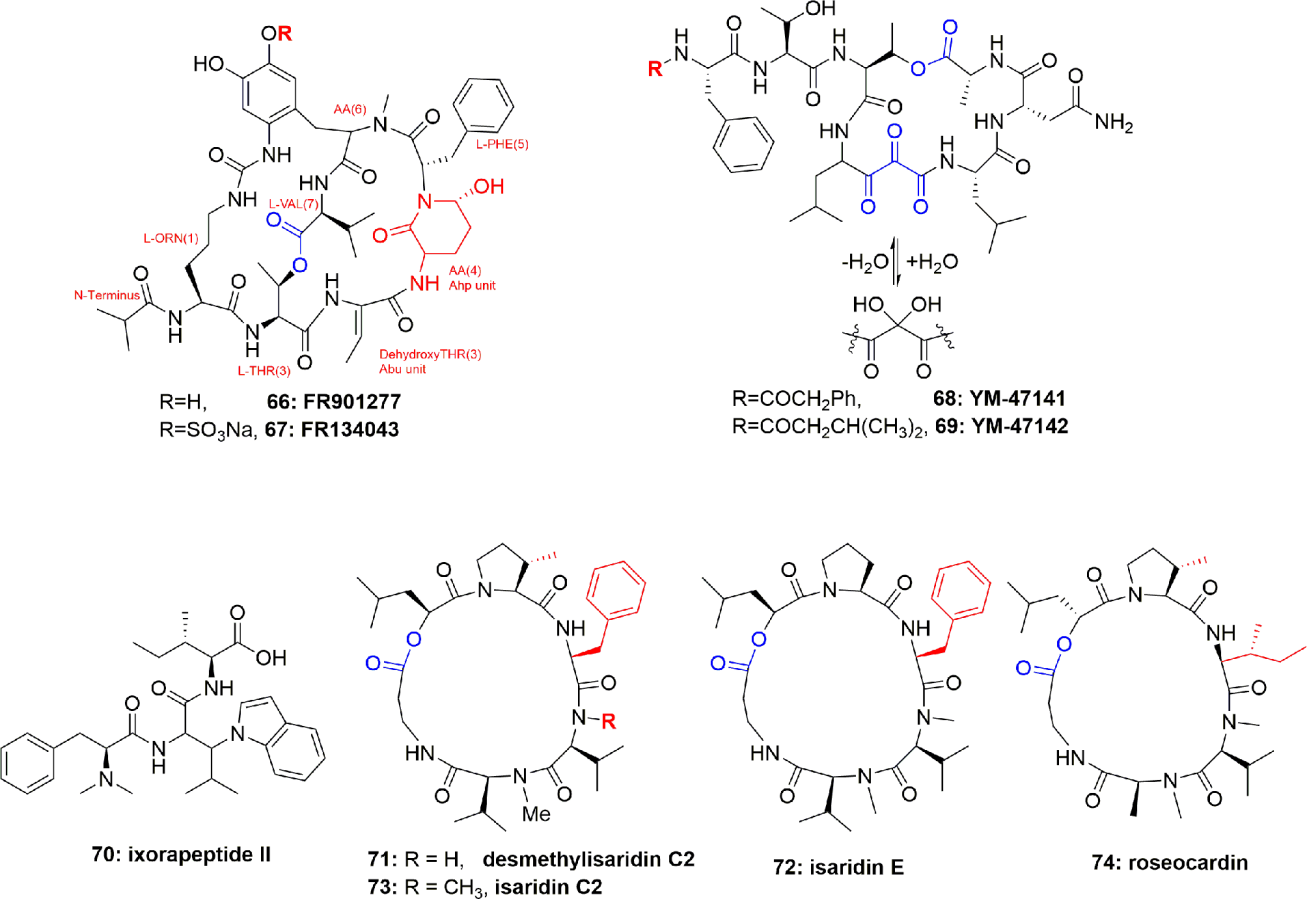
Structures of FR901277 (**66**), FR134043 (**67**), YM-47141 (**68**), YM-47142 (**69**); structure of the peptide ixorapeptide II (**70**) of *Ixora coccinea* (Rubiaceae), and structures of desmethylisaridin C2 (**71**), isaridin E (**72**), isaridin C2 (**73**), and roseocardin (**74**).

FR901277 (**66**) (**Figure 15**), a novel and unique bicyclic macrocyclic natural polypeptide, obtained from the bacterium filtrate *Streptomyces resistomicificus*, is a potent inhibitor of both PPE and HLE. Its unique bicyclic structure comprises of four normal aa [L-Orn(1), L-Thr(2), L-Phe(5), and L-Val(7)] and three unusual aa [dehydroxyThr(3), AA(4), and AA(6)], with a N-terminal of isopropyl carbonyl (Nakanishi et al., 2000b). FR901277 (**66**) inhibited both HLE and PPE with IC_50_ of 0.2 and 0.3 µM, respectively (Nakanishi et al., 2000b). FR134043 (**67**) (**Figure 15**) is a disulfonated semisynthetic derivative of **66**, which also has potent inhibitory activity against HLE with IC_50_ = 0.04 µM (Nakanishi et al., 2000a).

### Polypeptides of the Bacterium *Flexibacter* sp. Q17897

The two cyclic-depsipeptides, designated YM-47141 (**68**) and YM-47142 (**69**) (**Figure 15**), were isolated from the fermented bacterium *Flexibacter* sp. Q17897. Both peptides are considered as the first natural compounds containing vicinal tricarbonyl functionality. They showed potent HLE inhibition with IC_50_ values of 1.5 µM and 3.0 µM, respectively (Orita et al., 1995; Yasumuro et al., 1995). Their total synthesis has also been reported (Wasserman et al., 1999; Wasserman et al., 2000).

## Anti-elastase Polypeptide of Plant Origin

The peptide ixorapeptide II (**70**) (**Figure 15**) was discovered in the MeOH extract of *Ixora coccinea* (Rubiaceae). Compound **70** has been identified as a promising anti-inflammatory agent because it inhibited elastase release with IC_50_ value 5.6 µM. The important feature is that compound **70** exhibited a 73-fold more potent inhibition of elastase release than the commercial drug phenylmethylsulfonyl fluoride (PMSF). It was, therefore, concluded that peptide **70** could be a promising anti-inflammatory drug lead with no cytotoxicity, and may be subjected to *in vivo* studies and clinical trials (Lee et al., 2010).

## Anti-elastase Polypeptides of Fungal Origin

Besides cyanobacterial sources, epigenetic tools have also been utilized to get elastase inhibitor peptides from fungi. For example, supplementation of suberoylanilide hydroxamic acid (SAHA) to the medium of the fungus *Beauveria felina* resulted in the isolation of the cyclodepsipeptides of the isaridin type: desmethylisaridin C2 (**71**), isaridin E (**72**), isaridin C2 (**73**), and roseocardin (**74**) (**Figure 15**). Their structural elucidation was proven through extensive spectroscopic measurements and chemical derivatization and compared with the literature data, however, the sequence of the amino acids were established due to EIMS experiments. Compounds **71**-**74** inhibited FMLP-induced elastase release in human neutrophils with IC_50_ between 10.0 and 12.0 μM. SAR studies showed that the existence of allylic moiety in the co-isolated compounds destruxin A, resetoxin B, resulted in a decrease in anti-inflammatory activity. **71**-**74** exhibited anti-inflammatory activity without toxicity toward human neutrophils which were confirmed by the results of the measurement of cell viability by LDH which showed that these compounds hadn`t increased LDH release compared to the control (Chung et al., 2013).

The peptides AFUEI and AFLEI were isolated from *Aspergillus fumigatus* and *A*. *flavus*, respectively. They have an identical aa sequence. AFUEI is composed of 68 aa residues, and it was predicted as a signal peptide. AFUEI has a promising elastase inhibition which is more potent than the other elastase inhibitors in clinical trials (Sakuma et al., 2013).

## Anti-elastase Polypeptides of Animal Origin

Serine protease inhibitors (serpins) constitute a large protein class of native serine protease inhibitors with members spread over prokaryotes and eukaryotes (Zhang et al., 2017). Serpins have also been found in the venom of snakes, spiders, scorpions, cone snails, cnidarians, hymenopterans, and platypuses (Yuan et al., 2008). These inhibitors are mostly Kunitz-type toxins (the motif of the Kunitz-type toxins usually has a peptide chain of around 60 aa residues and is stabilized by three disulphide bridges (Yuan et al., 2008)). However, extensive transcriptomic and proteomic analysis of the venoms of animals also led to the discovery of non-Kunitz inhibitors (Xu et al., 2014; Liu et al., 2015). In addition, other polypeptides as elastase inhibitors have also been found in other animal sources. For example, elafin (skin-derived antileukoproteinase) was obtained from the horny layers of the human skin of patients with psoriasis where their skin was characterized by hyperproliferatin keratinocytes, and an inflammatory infiltrate consisting partly of neutrophils migrating into the affected skin epidermis. Elafin potently inhibited HLE and PPE in a 1:1 molar ratio with equilibrium dissociation constants (*Ki*) of 6 x 10^-10^ and 1 x 10^-9^ M, respectively. The aa sequencing revealed that elafin consists of 57 aa. Elafin was reported to have a crucial role in preventing elastase-mediated tissue proteolysis, which was attributed to the high affinity and the apparent specificity for elastases (Wiedow et al., 1990).

The elastasin peptide, identified in goats, is a serpin related to human α1-anti-chymotrypsin. Elastasin was an inhibitor of neutrophil elastase (k_ass._ = 1.5 x 10^6^ M^-1^.S^-1^). The specific activity, resistance to oxidative and proteolytic inactivation, and the presence of a P1 leucine residue in elastasin, is unique among inhibitory serpins. This serpin seems to be the major elastase inhibitor in goat plasma, which is involved in the control of goat neutrophil elastase (Potempa et al., 1995).

Pnserpin, a putative serpin from the thermophile *Pyrobaculum neutrophilum*, irreversibly inhibited elastase-like protease in a temperature range between 20 and 100°C and the inhibitory activity of pnserpin increased with the temperature (Zhang et al., 2017).

AvKTI, comprising of 170 aa, is the first spider (*Araneus ventricosus*) serine protease inhibitor of Kunitz-type, obtained from the body of the spider instead of its venom. Out of 170 aa, 19-aa comprise a signal peptide, 94-aa make pro-peptide, and a Kunitz domain consisting of 57-aa peptide that shows features matching to Kunitz-type inhibitors, including a P1 lysine site and six conserved cysteine residues; it inhibited HNE IC_50_: 0.447 µM (Wan et al., 2013b).

AvCI is a spider (*Araneus ventricosus*) polypeptide, consisted of 86 aa, including a 16-aa signal peptide and a 70-aa mature peptide that displays a P1 lysine residue and eight conserved cysteine residues. AvCI potently inhibited HNE with IC_50_ of 0.011 µM which was 1.65-fold more than its PPE inhibition (IC_50_ = 0.019 µM) (Wan et al., 2013a).

Kim et al. reported the first bee-derived serine protease inhibitor, AcCI, which was obtained from the body and venom of Asiatic honeybee (*Apis cerana*) worker bees. AcCI was found to consist of 85-aa that includes a 20-aa signal peptide and a 65-aa mature peptide that displays a P1 site and ten cysteine residues. AcCI inhibited HNE (IC_50_: 0.038 µM) which was a 1.8-fold stronger inhibition than that against PPE (IC_50_: 0.07 µM) (Kim et al., 2013).

BmKPI is the *Buthus martensi* Kunitz-type protease inhibitor which was present in the venom gland of the scorpion *B. martensi*.It has a unique disulfide framework where it has a unique cysteine skeleton reticulated by four disulfide bridges (three disulfide bridges in many other Kunitztype proteins). The functionally expressed recombinant BmKPI peptide showed potent inhibition activity against PPE (*Ki* 1.6 x 10^-7^ M) and it is the first functionally characterized Kunitz-type elastase inhibitor derived from scorpion venom. The unique disulfide bridge Cys53–Cys61 had little effect on its elastase inhibition as shown by cysteine mutagenesis experiment (Chen et al., 2013).

Bungaruskunin is a novel serine protease inhibitor and was isolated from the venom of *Bungarus fasciatus*. The predicted precursor is composed of 83 aa residues and it has a moderate inhibitory activity against elastase (*Ki* of 6.9 x 10^-4^ M) (Lu et al., 2008).

## Discussion

Inhibition of elastase enzyme is an important strategy for the alleviation of different inflammatory ailments and the discovery of new elastase modulators is deemed to be significant for the development of potential therapeutics and pharmacological tools. Polypeptides are an important class of natural products, identified as potential inhibitors of elastase enzyme, and hence can be used as a scaffold for designing more selective and potent inhibitors of the enzyme. Marine organisms are increasingly a prolific source of bioactive and unusual structural compounds (Donia and Hamann, 2003; Elsebai et al., 2011b; Elsebai et al., 2011a; Elsebai et al., 2014; Elsebai et al., 2016) and the cyanobacteria of marine origin have been recognized as a fruitful arsenal of polypeptides possessing promising inhibitory activity against serine proteases. Several cyclic peptides and depsipeptides have recently been isolated from cyanobacteria, and they have an attractive molecular architecture with a constrained conformation.

Nearly all the aforementioned polypeptides are cyclic depsi-peptides containing modified and unusual aa residues, such as the modified glutamic acid moiety, Ahp (3-amino-6-OH-2-piperidone). The Ahp-containing depsipeptides have the ability to inhibit serine proteases (such as elastase, chymotrypsin, and trypsin with different inhibition selectivity depending on the differences in their aa composition), and hence their ecological role is supposed to be as inhibitors of digestive enzymes and a chemical defense against crustacean predators. They exhibited significant anti-elastase activities even up to a nanomolar concentration, specially lyngbyastatin 4 (**1**) and its analogues such as lyngbyastatins 5-7 (**2-4**) and insulapeptolides C and D (**37**, **38**).

The Ahp-bearing cyclic depsi-peptides are a significant family, primarily due to their structural diversity, predominance, and potent protease-inhibitor activity. The role of such compounds in nature may be as digestive inhibitors in herbivores, feeding deterrents, and possibly regulators of the biosynthesis of coexisting secondary constituents (Salvador et al., 2013). Further SAR analysis of compounds **2-5** revealed that the Abu moiety plays a significant role in selective inhibition of elastase, and overall the cyclic structural core for **1-5** demonstrated a potent inhibitor prototype. The crystal structural data of the Abu-containing bicyclic inhibitor FR901277 (**66**) bound to PPE (Nakanishi et al., 2000b) established that the ethylidene functionality of Abu was stabilized by CH/π interaction (Salvador et al., 2013). It is therefore proposed that such an enzyme-inhibitor interaction may also exist in the case of monocyclic inhibitors **1-5**. The complexity and molecular diversity of polypeptides are evident from the lines above, which might be responsible for their anti-inflammatory potential. Structural complexity and diversity, however, is not the only reason; an important additional feature is their selectivity and specificity based on their mechanisms of action.

The selectivity inhibition of Ahp-containing depsi-peptides against elastase is increased when the Abu residue is neighbor to the Ahp residue as compared to other serine proteases. The reason for this varying activity could be the different binding preferences to the specificity pocket of the enzyme (Kuo et al., 2013). Previously, several reports have been published on the related Ahp-containing protease inhibitors of cyanobacterial origin, and are assumed to be enzyme-substrate mimics (Itou et al., 1999; Yamaki et al., 2005). This assumption leads to the conclusion that polypeptides also inhibit elastase in a competitive way using Michaelis–Menten kinetics, since the residue between Thr and Ahp units presumably defines the specificity toward specific serine proteases(Matern et al., 2003b; Yamaki et al., 2005; Salvador et al., 2013). Through identification of the IFR (interface forming residues) for serine proteases *in silico* docked to different inhibitors, it was concluded that the serine proteases interfaces prefer polar residues (with some exceptions)(Ribeiro et al., 2010).

Based on the current review, cyanobacteria are an abundant source of bioactive peptides and depsipeptides since they have been identified to produce a variety of similar anti-inflammatory polypeptides (Tan, 2010). It is, therefore, concluded that cyanobacteria could be one of the most promising and potential targets for drug discovery and development (Burja et al., 2001). Literature reports revealed that the cyanobacterial secondary metabolites are remarkably diverse in their structural features, with modified peptide–polyketide hybrids. They are biosynthesized by either nonribosomal polypeptide synthetases (NRPS), mixed polyketide synthase–NRPS pathways (Tan, 2007), or ribosomally (McIntosh et al., 2009).

Animal biological fluids and venoms are promising for their potential, and yet remain underestimated sources for biological agents against elastases. Animal venoms will potentiate the development of natural therapeutic and diagnostic drugs for human diseases that target different proteases.

Despite the fact that during the recent past, the synthetic libraries were being considered for the development of new lead drugs, we still believe that nature will continue to be a significant inspiring source of new anti-inflammatory drugs.

It is worth mentioning that the activity of the aforementioned polypeptides is lacking *in vivo* evaluation, however, the above-mentioned *in vitro* results may lead to *in vivo* studies of these molecules. Since most of these polypeptides are complex structures, which are difficult to synthesize chemically, to study their biological properties also requires screening of natural molecules. However, this is not the case for small molecules acting as elastase inhibitors (Saleem et al., 2018). Therefore, the *in vitro* elastase inhibitory studies of the natural polypeptides embodied in this document may provide a milestone for drug development and design.

## Author Contributions

SA outlined the article and collection of data. MS: collection of data, redrawing the structures, and proof-reading. NR and YL: proofreading. RD, AB, AN, and DA: proofreading and partial contribution for publication fees. ME: collection of data, redrawing the chemical structures, and proofreading.

## Conflict of Interest

The authors declare that the research was conducted in the absence of any commercial or financial relationships that could be construed as a potential conflict of interest.

## References

[B1] Adiv S. Aharonv-nadborny R. Carmeli S. (2010). Micropeptins from Microcystis aeruginosa collected in Dalton reservoir, Israel. Tetrahedron 66, 7429–7436. 10.1016/j.tet.2010.06.071

[B2] Barnes P. J. Stockley R. A. (2005). COPD: Current therapeutic interventions and future approaches. Eur. Respir. J. 25, 1084–1106. 10.1183/09031936.05.00139104 15929966

[B3] Burja A. M. Banaigs B. Abou-mansour E. Grant J. Wright P. C. (2001). Marine cyanobacteria‒ A prolific source of natural products. Tetrahedron Lett. 57, 9347–9377. 10.1016/S0040-4020(01)00931-0

[B4] Chen Z. Cao Z. Li W. Wu Y. (2013). Cloning and characterization of a novel Kunitz-type inhibitor from scorpion with unique cysteine framework. Toxicon 72, 5–10. 10.1016/j.toxicon.2013.05.022 23747274

[B5] Chung Y.-M. El-Shazly M. Chuang D.-W. Hwang T.-L. Asai T. Oshima Y. (2013). Suberoylanilide Hydroxamic Acid, a Histone Deacetylase Inhibitor, Induces the Production of Anti-inflammatory Cyclodepsipeptides from Beauveria felina. J. Nat. Prod. 76, 1260–1266. 10.1021/np400143j 23822585

[B6] Crocetti L. Quinn M. T. Schepetkin I. A. Giovannoni M. P. (2019). A patenting perspective on human neutrophil elastase (HNE) inhibitors, (2014-2018) and their therapeutic applications. Expert Opin. Ther. Pat. 29, 555–578. 10.1080/13543776.2019.1630379 31204543 PMC9642779

[B7] De M. V. Silva P. L. Macedo P. R. (2016). Animal Models of Chronic Obstructive Pulmonary Disease Exacerbations: A Review of the Current Status Abstract. J. Biomed. Sci. 5, 1–14. 10.4172/2254-609X.100022

[B8] Di L. (2014). Strategic approaches to optimizing peptide ADME properties. AAPS J. 17, 134–143. 10.1208/s12248-014-9687-3 25366889 PMC4287298

[B9] Dittrich A. S. Kuhbandner I. Gehrig S. Rickert-Zacharias V. Twigg M. Wege S. (2018). Elastase activity on sputum neutrophils correlates with severity of lung disease in cystic fibrosis. Eur. Respir. J. 51 (3), 1701910. 10.1183/13993003.01910-2017 29545279

[B10] Donia M. Hamann M. T. (2003). Marine natural products and their potential applications as anti-infective agents. Lancet Infect. Dis. 3, 338–348. 10.1016/S1473-3099(03)00655-8 12781505 PMC7106398

[B11] Elsebai M. F. Kehraus S. Lindequist U. Sasse F. Shaaban S. Guetschow M. (2011a). Antimicrobial phenalenone derivatives from the marine-derived fungus Coniothyrium cereale. Org. Biomol. Chem. 9, 802–808. 10.1039/C0OB00625D 21103541

[B12] Elsebai M. F. Natesan L. Kehraus S. Mohamed I. E. Schnakenburg G. Sasse F. (2011b). HLE-Inhibitory Alkaloids with a Polyketide Skeleton from the Marine-Derived Fungus Coniothyrium cereale. J. Nat. Prod. 74, 2282–2285. 10.1021/np2004227 21923104

[B13] Elsebai M. F. Nazir M. Kehraus S. Egereva E. Ioset K. N. Marcourt L. (2012). Polyketide skeletons from the marine alga-derived fungus coniothyrium cereale. Eur. J. Org. Chem. 2012 (31), 6197–6203. 10.1002/ejoc.201200700

[B14] Elsebai M. F. Saleem M. Tejesvi M. V. Kajula M. Mattila S. Mehiri M. (2014). Fungal phenalenones: chemistry, biology, biosynthesis and phylogeny. Nat. Prod. Rep. 31, 628–645. 10.1039/c3np70088g 24686921

[B15] Elsebai M. F. Ghabbour H. A. Mehiri M. (2016). Unusual Nitrogenous Phenalenone Derivatives from the Marine-Derived Fungus Coniothyrium cereale. Molecules 21, 1–13. 10.3390/molecules21020178 PMC6273853 26840293

[B16] Fujii K. Sivonen K. Naganawa E. Harada K. (2000). Non-toxic peptides from toxic cyanobacteria, Oscillatoria agardhii. Tetrahedron 56, 725–733. 10.1016/S0040-4020(99)01017-0

[B17] Giacalone V. D. Margaroli C. Mall M. A. Tirouvanziam R. (2020). Neutrophil Adaptations upon Recruitment to the Lung: New Concepts and Implications for Homeostasis and Disease. Int. J. Mol. Sci. 21, 851. 10.3390/ijms21030851 PMC7038180 32013006

[B18] Grach-pogrebinsky O. Sedmak B. Carmeli S. (2003). Protease inhibitors from a Slovenian Lake Bled toxic waterbloom of the cyanobacterium Planktothrix rubescens. Tetrahedron 59, 8329–8336. 10.1016/j.tet.2003.09.006

[B19] Gunasekera S. P. Miller M. W. Kwan J. C. Luesch H. Paul V. J. Gaines V. (2010). Molassamide, a Depsipeptide Serine Protease Inhibitor from the Marine Cyanobacterium. J. Nat. Prod. 73, 459–462. 10.1021/np900603f 20020755

[B20] Hilhorst M. van Paassen P. Tervaert J. W. C. Registry L. R. (2015). Proteinase 3-ANCA Vasculitis versus Myeloperoxidase-ANCA Vasculitis. J. Am. Soc. Nephrol. 26, 2314–2327. 10.1681/ASN.2014090903 25956510 PMC4587702

[B21] Horwitz M. Benson K. F. Person R. E. Aprikyan A. G. Dale D. C. (1999). Mutations in ELA2, encoding neutrophil elastase, define a 21-day biological clock in cyclic haematopoiesis. Nat. Genet. 23, 433–436. 10.1038/70544 10581030

[B22] Itou Y. Ishida K. Shin H. J. Murakami M. (1999). Oscillapeptins A to F, Serine Protease Inhibitors from the Three Strains of Oscillatoria agardhii. Tetrahedron 55, 6871–6882. 10.1016/S0040-4020(99)00341-5

[B23] Kang H. Krunic A. Orjala J. (2012). Stigonemapeptin, an Ahp-Containing Depsipeptide with Elastase Inhibitory Activity from the Bloom-Forming Freshwater Cyanobacterium Stigonema sp. J. Nat. Prod. 75, 807–811. 10.1021/np300150h 22483033 PMC3338906

[B24] Khan M. A. Ali Z. S. Sweezey N. Grasemann H. Palaniyar N. (2019). Progression of Cystic Fibrosis Lung Disease from Childhood to Adulthood: Neutrophils, Neutrophil Extracellular Trap (NET) Formation, and NET Degradation. Genes (Basel). 10, E183. 10.3390/genes10030183 30813645 PMC6471578

[B25] Kim B. Y. Lee K. S. Wan H. Zou F. M. Choi Y. S. Yoon H. J. (2013). Anti-elastolytic activity of a honeybee (Apis cerana) chymotrypsin inhibitor. Biochem. Biophys. Res. Commun. 430, 144–149. 10.1016/j.bbrc.2012.11.056 23200835

[B26] Kuo W. L. Chung C. Y. Hwang T. L. Chen J. J. (2013). Biphenyl-type neolignans from Magnolia officinalis and their anti-inflammatory activities. Phytochemistry 85, 153–160. 10.1016/j.phytochem.2012.08.014 23017219

[B27] Kwan J. C. Taori K. Paul V. J. Luesch H. (2009). Lyngbyastatins 8–10, Elastase Inhibitors with Cyclic Depsipeptide Scaffolds Isolated from the Marine Cyanobacterium Lyngbya semiplena. Mar. Drugs 7, 528–538. 10.3390/md7040528 20098596 PMC2810234

[B28] Lee C. Liao Y. Hwang T. Wu C. Chang F. (2010). Ixorapeptide I and ixorapeptide II , bioactive peptides isolated from Ixora coccinea. Bioorg. Med. Chem. Lett. 20, 7354–7357. 10.1016/j.bmcl.2010.10.058 21106454

[B29] Liu H. Chen J. Wang X. Yan S. Xu Y. San M. (2015). Functional characterization of a new non-Kunitz serine protease inhibitor from the scorpion Lychas mucronatus. Int. J. Biol. Macromol. 72, 158–162. 10.1016/j.ijbiomac.2014.08.010 25150597

[B30] Lodin-Friedman A. Carmeli S. (2013). Metabolites from Microcystis aeruginosa Bloom Material Collected at a Water Reservoir near Kibbutz Hafetz Haim, Israel. J. Nat. Prod. 76, 1196–1200. 10.1021/np400281q 23718637

[B31] Lu J. Yang H. Yu H. Gao W. Lai R. Liu J. (2008). A novel serine protease inhibitor from Bungarus fasciatus venom. Peptides 29, 369–374. 10.1016/j.peptides.2007.11.013 18164783

[B32] Luo D. Luesch H. (2020). Ahp-Cyclodepsipeptide Inhibitors of Elastase: Lyngbyastatin 7 Stability, Scalable Synthesis, and Focused Library Analysis. ACS Med. Chem. Lett. 11, 419–425. 10.1021/acsmedchemlett.9b00473 32292544 PMC7153021

[B33] Matern U. Oberer L. Falchetto R. A. Erhard M. Ko W. A. Herdman M. (2001). Scyptolin A and B, cyclic depsipeptides from axenic cultures of Scytonema hofmanni PCC 7110. Phytochemistry 58, 1087–1095. 10.1016/S0031-9422(01)00400-9 11730873

[B34] Matern U. Oberer L. Falchetto R. A. Erhard M. Ko W. A. Herdman M. (2003a). Corrigendum to ‘“ Scyptolin A and B , cyclic depsipeptides from axenic cultures of Scytonema hofmanni PCC 7110 “‘ [Phytochemistry 58 (2001 ) 1087 – 1095 ] §. Phytochemistry 64, 1175. 10.1016/j.phytochem.2003.08.015 11730873

[B35] Matern U. Schleberger C. Jelakovic S. Weckesser J. Schulz G. E. (2003b). Binding Structure of Elastase Inhibitor Scyptolin A. Chem. Biol. 10, 997–1001. 10.1016/j.chembiol.2003.10.001 14583266

[B36] Matthew S. Ross C. Rocca J. R. Paul V. J. Luesch H. Chemistry M. (2007). Lyngbyastatin 4, a Dolastatin 13 Analogue with Elastase and Chymotrypsin Inhibitory Activity from the Marine Cyanobacterium Lyngbya confervoides. J. Nat. Prod. 70, 124–127. 10.1021/np060471k 17253864

[B37] Matthew S. Paul V. J. Luesch H. (2009a). Largamides A - C, tiglic acid-containing cyclodepsipeptides with elastase-inhibitory activity from the marine cyanobacterium Lyngbya confervoides. Planta Med. 75, 528–533. 10.1055/s-0029-1185332 19214948 PMC2748991

[B38] Matthew S. Paul V. J. Luesch H. (2009b). Tiglicamides A–C, cyclodepsipeptides from the marine cyanobacterium Lyngbya confervoides. Phytochemistry 70, 2058–2063. 10.1016/j.phytochem.2009.09.010 19815244 PMC2787822

[B39] McDonough M. A. Schofield C. J. (2003). New structural insights into the inhibition of serine proteases by cyclic peptides from bacteria. Chem. Biol. 10, 898–900. 10.1016/j.chembiol.2003.10.002 14583255

[B40] McIntosh J. A. Donia M. S. Schmidt E. W. (2009). Ribosomal Peptide Natural Products: Bridging the Ribosomal and Nonribosomal Worlds. Nat. Prod. Rep. 26, 537–559. 10.1039/B714132G 19642421 PMC2975598

[B41] Mehner C. Müller D. Kehraus S. Hautmann S. Gütschow M. Konig G. M. (2008). New Peptolides from the Cyanobacterium Nostoc insulare as Selective and Potent Inhibitors of Human Leukocyte Elastase. ChemBioChem 9, 2692–2703. 10.1002/cbic.200800415 18924217

[B42] Murakami M. Sun Q. Ishida K. Matsuda H. Okino T. Yamaguchi K. (1997). Microviridins, elastase inhibitors from the cyanobacterium Nostoc minutum (NIES-26). Phytochemistry 45, 1197–1202. 10.1016/S0031-9422(97)00131-3

[B43] Nakanishi I. Fujikawa A. Imai K. Sato A. (2000a). 1H-NMR determination of the solution structure and absolute configuration of FR134043, a novel inhibitor of human leukocyte elastase. J. Pept. Res. 55, 120–128. 10.1034/j.1399-3011.2000.00165.x 10784028

[B44] Nakanishi I. Kinoshita T. Sato A. Tada T. (2000b). Structure of Porcine Pancreatic Elastase Complexed with FR901277, a Novel Macrocyclic Inhibitor of Elastases, at 1.6 Å Resolution. Biopolymers 53, 434–445. 10.1002/(SICI)1097-0282(20000415)53:5<434::AID-BIP7>3.0.CO;2-5 10738204

[B45] Okino T. Matsuda H. Murakami M. Yamaguchi K. (1995). New Microviridins, Elastase Inhibitors from the Blue-Green-Alga Microcystis-Aeruginosa. Tetrahedron 51, 10679–10686. 10.1016/0040-4020(95)00645-O

[B46] Okino T. Qi S. Matsuda H. Murakami M. Yamaguchi K. (1997). Nostopeptins A and B, Elastase Inhibitors from the Cyanobacterium Nostoc minutum. J. Nat. Prod. 60, 158–161. 10.1021/np960649a

[B47] Orita M. Yasumuro K. Kokubo K. Shimizu M. Abe K. Tokunaga T. (1995). YM-47141 and YM-47142, new elastase inhibitors produced by Flexibacter sp. Q17897. II. Structure elucidation. J. Antibiot. (Tokyo). 48, 1430–1434. 10.7164/antibiotics.48.1430 8557599

[B48] Potempa J. Enghildt J. J. Travist J. (1995). The primary elastase inhibitor (elastasin ) and trypsin inhibitor (contrapsin ) in the goat are serpins related to human α1-anti-chymotrypsin. Biochem. J. 306, 191–197. 10.1042/bj3060191 7864809 PMC1136500

[B49] Ribeiro C. Togawa R. C. Neshich I. A. P. Mazoni I. Mancini A. L. Minardi R. C. (2010). Analysis of binding properties and specificity through identification of the interface forming residues (IFR) for serine proteases in silico docked to different inhibitors. BMC Struct. Biol. 10, 36. 10.1186/1472-6807-10-36 20961427 PMC2974730

[B50] Rubio B. K. Parrish S. M. Yoshida W. Schupp P. J. Schils T. Williams P. G. (2010). Depsipeptides from a Guamanian marine cyanobacterium , Lyngbya bouillonii, with selective inhibition of serine proteases. Tetrahedron Lett. 51, 6718–6721. 10.1016/j.tetlet.2010.10.062 21103388 PMC2987581

[B51] Sakuma M. Imada K. Okumura Y. Uchiya K. Yamashita N. Ogawa K. (2013). X-ray structure analysis and characterization of AFUEI, an elastase inhibitor from Aspergillus fumigatus. J. Biol. Chem. 288, 17451–17459. 10.1074/jbc.M112.433987 23640894 PMC3682545

[B52] Saleem M. Nazir M. Hussain H. Tousif M. I. (2018). Natural Phenolics as Inhibitors of the Human Neutrophil Elastase (HNE) Release: An Overview of Natural Anti-inflammatory Discoveries during Recent Years. Antiinflamm. Antiallergy. Agents Med. Chem. 17, 70–94. 10.2174/1871523017666180910104946 30198444

[B53] Salvador L. A. Taori K. Biggs J. S. Jakoncic J. Ostrov D. A. Paul V. J. (2013). Potent Elastase Inhibitors from Cyanobacteria: Structural Basis and Mechanisms Mediating Cytoprotective and Anti-Inflammatory Effects in Bronchial Epithelial Cells. J. Med. Chem. 56, 1276–1290. 10.1021/jm3017305 23350733 PMC3624605

[B54] Shin H. J. Murakami M. Matsuda H. Ishida K. Yamaguchi K. (1995). Oscillapeptin, an Elastase and Chymotrypsin Inhibitor from the Cyanobacterium Oscillatoria agardhii (NIES-204). Tetrahedron Lett. 36, 5235–5238. 10.1016/00404-0399(50)0980Q-

[B55] Sisay M. T. Hautmann S. Mehner C. König G. M. Bajorath J. Gütschow M. (2009). Inhibition of Human Leukocyte Elastase by Brunsvicamides A–C: Cyanobacterial Cyclic Peptides. ChemMedChem 4, 1425–1429. 10.1002/cmdc.200900139 19569166

[B56] Tan L. T. (2007). Bioactive natural products from marine cyanobacteria for drug discovery. Phytochemistry 68, 954–979. 10.1016/j.phytochem.2007.01.012 17336349

[B57] Tan L. T. (2010). Filamentous tropical marine cyanobacteria: A rich source of natural products for anticancer drug discovery. J. Appl. Phycol. 22, 659–676. 10.1007/s10811-010-9506-x

[B58] Taori K. Matthew S. Rocca J. R. Paul V. J. Luesch H. (2007). Lyngbyastatins 5–7, Potent Elastase Inhibitors from Floridian Marine Cyanobacteria, Lyngbya spp. J. Nat. Prod. 70, 1593–1600. 10.1021/np0702436 17910513

[B59] Taori K. Paul V. J. Luesch H. April R. V. (2008). Kempopeptins A and B, Serine Protease Inhibitors with Different Selectivity Profiles from a Marine Cyanobacterium, Lyngbya sp. J. Nat. Prod. 71, 1625–1629. 10.1021/np8002172 18693761

[B60] Thomas M. P. Whangbo J. McCrossan G. Deutsch A. J. Martinod K. Walch M. (2014). Leukocyte protease binding to nucleic acids promotes nuclear localization and cleavage of nucleic acid binding proteins. J. Immunol. 192, 5390–5397. 10.4049/jimmunol.1303296 24771851 PMC4041364

[B61] Thulborn S. J. Mistry V. Brightling C. E. Moffitt K. L. Ribeiro D. Bafadhel M. (2019). Neutrophil elastase as a biomarker for bacterial infection in COPD. Respir. Res. 20, 170. 10.1186/s12931-019-1145-4 31362723 PMC6668103

[B62] Tsai Y.-F. Yu H.-P. Chang W.-Y. Liu F.-C. Huang Z.-C. Hwang T.-L. (2015). Sirtinol inhibits neutrophil elastase activity and attenuates lipopolysaccharide-mediated acute lung injury in mice. Sci. Rep. 5, 1–10. 10.1038/srep08347 PMC4322352 25666548

[B63] Wagner C. J. Schultz C. Mall M. A. (2016). Neutrophil elastase and matrix metalloproteinase 12 in cystic fibrosis lung disease. Mol. Cell. Pediatr. 3, 25. 10.1186/s40348-016-0053-7 27456476 PMC4960106

[B64] Wan H. Lee K. S. Kim B. Y. Yuan M. Zhan S. You H. (2013a). A spider (Araneus ventricosus) chymotrypsin inhibitor that acts as an elastase inhibitor and a microbial serine protease inhibitor. Comp. Biochem. Physiol. Part B 165, 36–41. 10.1016/j.cbpb.2013.03.004 23499942

[B65] Wan H. Lee K. S. Kim B. Y. Zou F. M. Yoon H. J. Je Y. H. (2013b). A Spider-Derived Kunitz-Type Serine Protease Inhibitor That Acts as a Plasmin Inhibitor and an Elastase Inhibitor. PloS One 8, 1–8. 10.1371/journal.pone.0053343 PMC3537671 23308198

[B66] Wasserman H. H. Chen J. Xia M. V Y. U. Box P. O. (1999). Total Syntheses of Depsipeptide Elastase Inhibitors YM-47141 and YM-47142 with use of Ylide Protection and Coupling Methods. J. Am. Chem. Soc 121, 1401–1402. 10.1021/ja9840302

[B67] Wasserman H. H. Chen J. H. Xia M. (2000). The chemistry of vicinal tricarbonyls: Total syntheses of elastase inhibitors YM-47141 and YM-47142. Helv. Chim. Acta 83, 2607–2616. 10.1002/1522-2675(20000906)83:9<2607::AID-HLCA2607>3.0.CO;2-B

[B68] Wiedow O. Schröder J.-M. Gregory H. Young J. A. Christophers E. (1990). Elafin: an elastase-specific inhibitor of human skin. Purification, characterization, and complete amino acid sequence. J. Biol. Chem. 265, 14791–14795. 2394696

[B69] Xu X. Duan Z. Di Z. He Y. Li J. Li Z. (2014). Proteomic analysis of the venom from the scorpion Mesobuthus martensii. J. Proteomics 106, 162–180. 10.1016/j.jprot.2014.04.032 24780724

[B70] Yamaki H. Sitachitta N. Sano T. Kaya K. (2005). Two New Chymotrypsin Inhibitors Isolated from the Cyanobacterium Microcystis aeruginosa NIES-88. J. Nat. Prod. 68, 14–18. 10.1021/np0401361 15679310

[B71] Yasumuro K. Suzuki Y. Shibazaki M. Teramura K. (1995). YM-47141 and 47142, new elastase inhibitors produced by Flexibacter sp. Q17897. I. Taxonomy, fermentation, isolation, physico-chemical properties and biological activities. J. Antibiot. (Tokyo). 48, 1425–1429. 10.7164/antibiotics.48.1425 8557598

[B72] Yuan C.-H. He Q.-Y. Peng K. Diao J.-B. Jiang L.-P. Tang X. (2008). Discovery of a distinct superfamily of Kunitz-type toxin (KTT) from tarantulas. PloS One 3, e3414. 10.1371/journal.pone.0003414 18923708 PMC2561067

[B73] Zafrir-ilan E. Carmeli S. (2010). Eight novel serine proteases inhibitors from a water bloom of the cyanobacterium. Tetrahedron 66, 9194–9202. 10.1016/j.tet.2010.09.067

[B74] Zeiher B. G. Artigas A. Vincent J.-L. Dmitrienko A. Jackson K. Thompson B. T. (2004). Neutrophil elastase inhibition in acute lung injury: results of the STRIVE study. Crit. Care Med. 32, 1695–1702. 10.1097/01.CCM.0000133332.48386.85 15286546

[B75] Zhang H. Fei R. Xue B. Yu S. Zhang Z. Zhong S. (2017). Pnserpin: A Novel Serine Protease Inhibitor from Extremophile Pyrobaculum neutrophilum. Int. J. Mol. Sci. 18, 113. 10.3390/ijms18010113 PMC5297747 28067849

